# Mitoguardin-2–mediated lipid transfer preserves mitochondrial morphology and lipid droplet formation

**DOI:** 10.1083/jcb.202207022

**Published:** 2022-10-25

**Authors:** Zhouping Hong, Jyoti Adlakha, Neng Wan, Emily Guinn, Fabian Giska, Kallol Gupta, Thomas J. Melia, Karin M. Reinisch

**Affiliations:** 1 Department of Cell Biology, Yale University School of Medicine, New Haven, CT; 2 Nanobiology Institute, Yale University, West Haven, CT; 3 Aligning Science Across Parkinson’s Collaborative Research Network, Chevy Chase, MD

## Abstract

Lipid transport proteins at membrane contacts, where organelles are closely apposed, are critical in redistributing lipids from the endoplasmic reticulum (ER), where they are made, to other cellular membranes. Such protein-mediated transfer is especially important for maintaining organelles disconnected from secretory pathways, like mitochondria. We identify mitoguardin-2, a mitochondrial protein at contacts with the ER and/or lipid droplets (LDs), as a lipid transporter. An x-ray structure shows that the C-terminal domain of mitoguardin-2 has a hydrophobic cavity that binds lipids. Mass spectrometry analysis reveals that both glycerophospholipids and free-fatty acids co-purify with mitoguardin-2 from cells, and that each mitoguardin-2 can accommodate up to two lipids. Mitoguardin-2 transfers glycerophospholipids between membranes in vitro, and this transport ability is required for roles both in mitochondrial and LD biology. While it is not established that protein-mediated transfer at contacts plays a role in LD metabolism, our findings raise the possibility that mitoguardin-2 functions in transporting fatty acids and glycerophospholipids at mitochondria-LD contacts.

## Teaser

Mitochondrial protein mitoguardin-2, at contacts with the ER or with lipid droplets (LDs), binds fatty acids and phospholipids, and non-specifically transfers phospholipids between membranes in vitro. Its lipid transfer ability is required for roles in mitochondrial and LD biology.

## Introduction

Membrane contacts, sites where organelles are closely apposed, have emerged as a major means of intraorganellar communication and regulation (Lees et al., 2017; Prinz et al., 2020). These sites are important for membrane lipid homeostasis, including for lipid transfer between organelles, and especially to/from organelles disconnected from vesicle trafficking pathways, such as mitochondria. Proteins localized to contacts solubilize and transfer lipids across the cytosol between organellar membranes. Some such proteins mediate bulk lipid transfer, for example for membrane expansion, while others transport specific lipids, such as phosphoinositide lipids, to tweak the lipid compositions of existing membranes (Reinisch and Prinz, 2021). One approach to better understanding the physiological roles of contact sites and the processes that occur there is to characterize protein residents of these sites.

**Figure 1.**
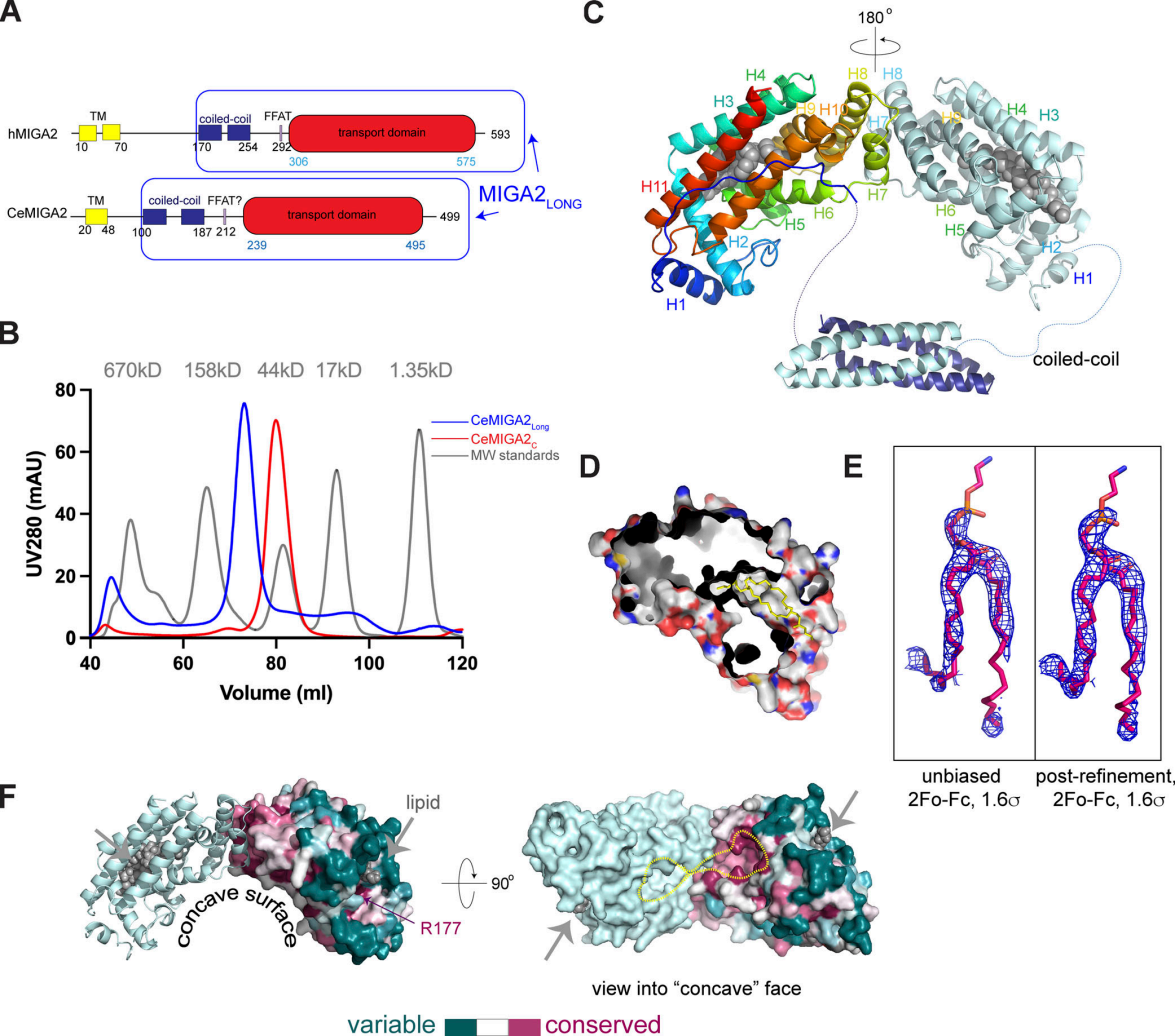
**The structure of MIGA2. (A)** Schematic of MIGA2 domain architecture. **(B)** Size exclusion profiles (Superdex 200 16/60) of CeMIGA2_long_ and CeMIGA2_C_, along with size standards (gray). CeMIGA2_long_ is a dimer, whereas CeMIGA2_C_ elutes more slowly as a monomer. hMIGA2 constructs behave the same (not shown). MW, molecular weight. **(C)** Ribbons diagram of the CeMIGA2_long_ dimer that was crystallized, including the lipid transport module (colored from blue at the N-terminus to red at the C-terminus in one copy) and the four-helix bundle that mediates dimerization. MIGA2_C_ dimerizes in the crystal as indicated. **(D)** MIGA2_C_ is cut away to reveal parts of the lipid binding tunnel. Residues at the MIGA2_C_ surface are colored red, blue, and white for oxygens, nitrogens, and carbons to illustrate that the tunnel is lined with hydrophobic residues. A yellow lipid is shown in the lipid binding site. **(E)** Initial electron density into which glycerophospholipid was modeled and the same density after refinement. Both densities are from 2Fo-Fc difference maps contoured at 1.6 σ. **(F)** Conservation analysis of MIGA2_C_ (calculated in Consurf [Ashkenazy et al., 2016], based on 150 sequences). A MIGA2 dimer is shown, and conservation is indicated for one of the monomers. Bound lipids are indicated. A strictly conserved arginine residue near the lipid headgroup, R177 in CeMIGA2, is indicated. The molecule is rotated to view the concave conserved surface former by the dimer. The “empty” end of the tunnels, where we did not find lipid density, are outlined in yellow. Additional lipids may be bound there. The interface between MIGA2_C_ domains, formed by helices H7 and H8, is also highly conserved.

Here, we characterize mitoguardin-2 (MIGA2), a mitochondrial protein present across most tissue types in higher eukaryotes, as a lipid transfer protein. MIGA2 localizes to contact sites between mitochondria and the ER or mitochondria and lipid droplets (LDs; Freyre et al., 2019). It comprises an N-terminal transmembrane segment, which anchors it in the outer mitochondrial membrane, followed by a coiled-coil embedded in a largely unstructured linker, and a folded module at its C-terminus (Fig. 1 A). An FFAT (two phenylalanines in an acidic tract) motif within the linker region of human MIGA2 binds the ER proteins VAP-A/B to promote the formation of mitochondrial–ER contacts (Freyre et al., 2019). The C-terminal portion of MIGA2 is reported to promote contacts to LDs (Freyre et al., 2019). MIGA2 plays a role in mitochondrial health as its lack leads to mitochondrial fragmentation (Zhang et al., 2016), and in mammals it is implicated in LD biogenesis (Freyre et al., 2019). It is involved in the differentiation of and de novo lipogenesis in white adipocytes, promoting triglyceride synthesis and LD formation by still unclear mechanisms (Freyre et al., 2019). We show that a channel in the C-terminal domain of MIGA2, lined by hydrophobic residues, enables this domain to solubilize lipids and to transfer them between membranes in vitro, and that this ability is essential for MIGA2 functions in vivo, including in both mitochondrial and LD biology.

## Results and discussion

### Its structure suggests MIGA2 as a lipid transfer protein

To analyze MIGA2’s function biochemically, we over-expressed in bacteria and purified cytosolic fragments of human and *Caenorhabditis elegans* MIGA2 comprising portions of the linker region and the C-terminal domain (hMIGA2_long_, CeMIGA_long_; Fig. 1 A) or only the C-terminal domain (hMIGA2_C_, CeMIGA_C_). The C-terminal domain migrated as a monomer as assessed by size exclusion chromatography, whereas the longer fragments that included a coiled-coil in the linker region dimerized (Fig. 1 B). Its propensity to dimerize can explain previous observations that MIGA2 overexpression causes mitochondrial clustering in cells (Zhang et al., 2016).

We crystallized CeMIGA_long_ (residues 106–496), including the coiled-coil segment in the linker region as well as the C-terminal domain, and determined its structure at 3.3 Å resolution. The crystals belonged to spacegroup P3_1_21. We used an AlphaFold2-generated model of CeMIGA2_C_ as a search model for phasing by molecular replacement, finding two dimers comprising four such modules in each asymmetric unit. The domains in the MIGA2_C_ dimer are related by a 180° rotation (Fig. 1 C). (Thus, in the context of the intact protein, MIGA2_C_ likely exists as a dimer, even though MIGA2C alone is monomeric by size exclusion chromatography.) Maps additionally showed density for two four-helix bundles, each corresponding to a dimer of the coiled-coils from the linker region (Fig. 1 C). Notably, the fold predicted for the MIGA2 C-terminal domain was not previously reported in the Protein Data Bank (PDB) but is consistent with experimental data as assessed by the success of the molecular replacement strategy (the root mean square deviation [RMSD] between the experimental and predicted structures is 0.58 Å over 241 Cα’s in CeMIGA2_C_).

**Figure S1.**
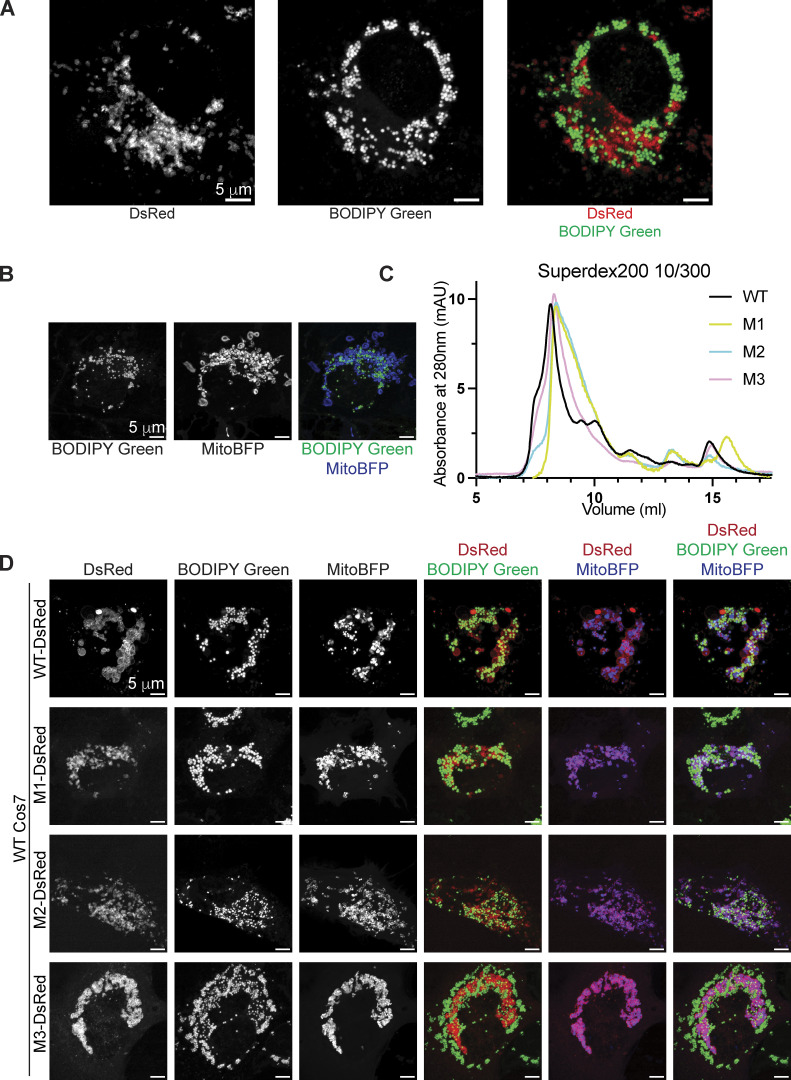
**Characterization of mutant MIGA2 constructs. (A)** Cos7 cells were transfected with MitoBFP and MIGA2(R334E/R337E/R422E/K481D)-DsRed bearing mutations in the conserved surface of MIGA2_C_ (with pLVX-CMV vector), treated with OA for 24 h, and imaged. MIGA2(R334E/R337E/R422E/K481D)-DsRed does not co-localize with LDs. **(B)** Cos7 cells transfected with MitoBFP were similarly treated and imaged. The morphology of mitochondria and LDs in WT Cos7 cell is shown. **(C)** Gel filtration profile of WT hMIGA2_long_ and mutants constructs M1, M2, M3 on a Superdex 200 10/30 column. The three mutant proteins elute as WT after the void volume, indicating that the mutants are not misfolded or aggregated. **(D)** Cos7 cells were transfected with MitoBFP and WT MIGA2-Dsred or MIGA2-DsRed M1, M2, or M3 transfer-impaired constructs (with pLVX-CMV vector), treated with OA for 24 h, and imaged. These constructs co-localize with mitochondria and LDs, as shown. Scale bar, 5 μm.

The all-helical C-terminal domain of MIGA2 is globular, measuring ∼65 × 35 × 35 Å, and features an L-shaped channel (∼895 Å^3^ in volume as calculated in CASTp [Tian et al., 2018]). The channel is lined with hydrophobic residues and so is suitable for solubilizing lipids (Fig. 1, C and D). As MIGA2 localizes to contact sites, like most lipid transporters, this finding suggested a function for MIGA2 as a lipid transfer protein. Poorly defined density, which was not modeled, occupies one end of the channel. Difference density reminiscent of a glycerophospholipid at the other end of the channel was modeled as such (Fig. 1 E). The bound lipid co-purified with the protein from the *Escherichia coli* expression host and presumably corresponds to a mixture of glycerophospholipids present in this bacterium (primarily phosphatidylethanolamine [PE] and phosphatidylglycerol [PG]). There was no density for the lipid headgroup, as might be expected if lipids are bound non-specifically. Residues within the channel were conserved evolutionarily (as analyzed by Consurf [Ashkenazy et al., 2016], based on 150 sequences), supporting their functional importance. Surface residues near the lipid headgroup (or where the lipid headgroup is expected to be) are highly variable, except for R177 (Fig. 1 F). R177 is strictly conserved across species and appears to be important for lipid binding (see analysis in “Its lipid transfer ability is essential for MIGA2 function in vivo”); likely through interaction with the glycerol backbone or headgroup of lipid ligands. The MIGA2_C_ domain, which was shown to be important for LD targeting (Freyre et al., 2019), dimerizes to form a concave surface patch that is conserved (Fig. 1 F). As the C-terminal portion of MIGA2 is required for its interactions with LDs (Freyre et al., 2019), the conserved patch could mediate these interactions (C-terminal helices proposed by Freyre et al. [2019] to be amphipathic and to mediate the interaction are, in fact, part of MIGA2_C_, corresponding to helices 9 and 10 in the structure; Fig. 1 C). Accordingly, a MIGA2 mutant in which residues in the conserved surface are mutated (R334E/R337E/R422E/K481D) does not co-localize with LDs (Fig. S1 A). The conserved surface of the MIGA2_C_ dimer is more highly curved than that of the comparatively flat LD, making a direct association between MIGA2_C_ and lipid surfaces unlikely. The MIGA2 conserved surface may instead interact with LD-associated proteins.

**Figure 2.**
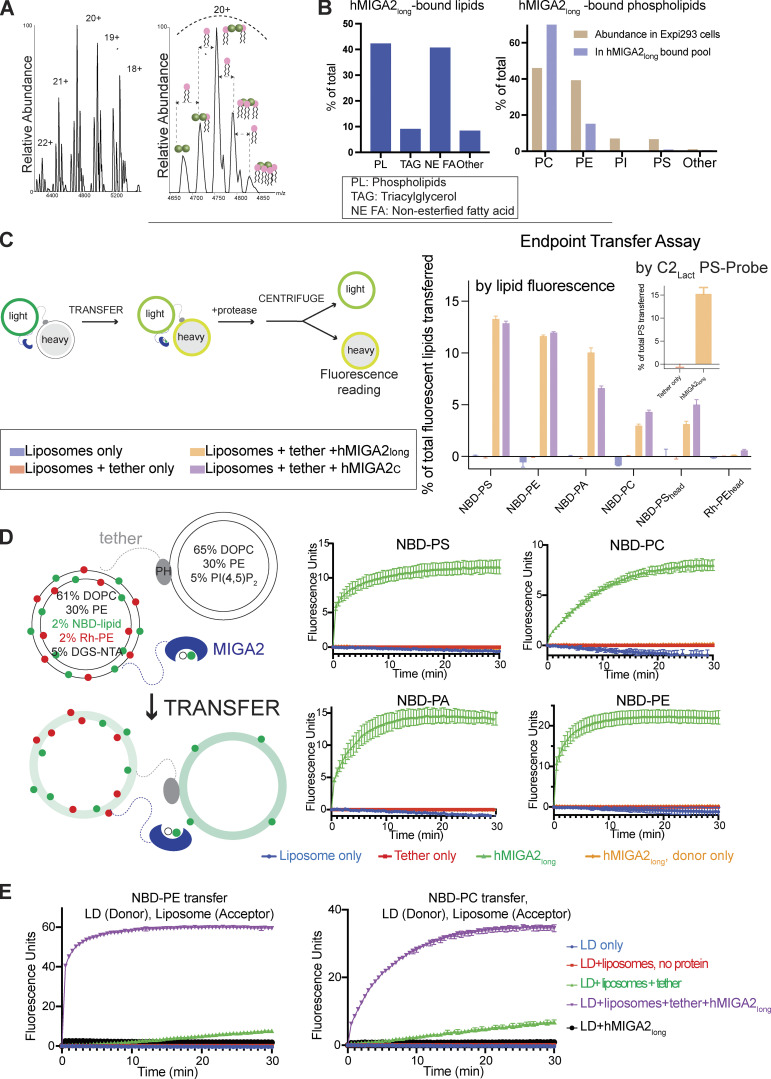
**MIGA2 binds and transfers lipids between membranes. (A)** Left: Native mass spectra of hMIGA2_long_-6xhis purified from bacteria, showing exclusive presence of the dimeric protein. Each charge state is further accompanied by a series of up to four lipid bound peaks. Right: Expansion of the 20 + charge state of native MS for hMIGA2_long_-6xhis dimer, showing up to four glycerophospholipids bound, with the average mass of 752 ± 5 Da. **(B)** Untargeted lipidomics of 3xFLAG-hMIGA2_long_, purified from mammalian (Expi293) cells. At left: Distribution of lipid classes bound to hMIGA2_long_. At right: Distribution of glycerophospholipids bound to hMIGA2_long_ as compared to their abundance in cells (Lees et al., 2017). **(C)** In the end-point transfer assay, hMIGA2 constructs are tethered between “heavy” acceptor liposomes (containing 0.75 M sucrose; lipid composition: 65% DOPC, 30% PE, and 5% PI[4,5]P_2_) and light donor liposomes containing a single species of fluorescent lipid (containing no sucrose; lipid composition: 63% DOPC, 30% PE, 2% NBD- or Rh-lipid, 5% DGS-NTA). After lipid transfer (after 30 min), proteinase K was added to digest proteins, the light donor and heavy acceptor liposomes were separated by centrifugation, and the fluorescence increase of the heavy acceptor liposomes monitored. Transferred lipid was quantitated based on liposome standards incorporating 2, 1, 0.5, 0.25, or 0% of the appropriate fluorescent lipid. Both hMIGA2_long_ and hMIGA2_C_ transport NBD-PE_acyl_, -PS_acyl_, -PA_acyl_, and NBD-PC_acyl_; NBD-PS_head_ is transferred to a lesser extent, and Rh-PE_head_ is not transferred, indicating that modification of the lipid headgroup can interfere with transport by MIGA2. In a similar experiment, we monitored transfer of unmodified PS using a PS-specific protein probe (C2-domain of lactadherin [Moser von Filseck et al., 2015]) to assess PS transfer. Donor liposomes initially contained 5% PS. Donor and acceptor liposomes were separated by addition of EDTA and imidazole rather than protease, so as not to destroy the PS-probe. Supernatant (containing donor liposomes) and pellet (acceptor liposomes) fractions, in the presence and absence of MIGA2, were analyzed by SDS-PAGE to quantitate the PS-probe associated with each fraction. Transfer efficiency of acyl chain modified NBD-PS, as monitored by fluorescence, is comparable to that of natural PS, indicating that the acyl chain modification does not interfere with transfer by MIGA2. **(D)** In the FRET-based transfer assay, donor and acceptor liposomes (compositions indicated) were tethered together in the presence or absence MIGA2 linked to the donor liposomes. The donor liposomes initially contain Rh-PE_head_ and NBD-lipids (-PS_acyl_, -PC_acyl_, -PA_acyl_, -PE_acyl_), where FRET between the Rh and NBD initially reduces NBD fluorescence. As lipids are transferred from donor to acceptor liposomes, the Rh- and NBD-labeled lipids are diluted, resulting in reduced FRET and an increase in NBD fluorescence. hMIGA2_long_ can transport NBD-PS_acyl_, NBD-PA_acyl_, NBD-PE_acyl_, and NBD-PC_acyl_. A donor only control shows that the fluorescence increase was due to transfer of lipids between liposomes rather than solely lipid extraction by hMIGA2. After the transfer reaction was completed, dithionite was added to rule out the possibility of fusion between donor and acceptor liposomes, which would also result in a fluorescence increase (Fig. S2 C). Each experiment was performed in triplicate. SDs are shown. NBD-PS_head_ transfer is less efficient (Fig. S2 D). **(E)** A similar FRET experiment was carried out to assess transfer from artificial LDs (rather than donor liposomes) to acceptor liposomes and shows that MIGA2 transfers NBD-PE_acyl_ and NBD-PC_acyl_ between the artificial LDs and liposomes. Positive stain transmission electron microscopy was used to assess the quality of the LD preparation (Fig. S2 E) as in Wang et al. (2016).

We analyzed hMIGA2_long_ (residues 170–575), similarly produced in bacteria, by native mass spectrometry (MS), finding that up to four lipids are bound per hMIGA2_long_ dimer, with masses corresponding to glycerophospholipid species present in *E. coli* (primarily PE and PG; Fig. 2 A). While we can unambiguously observe one lipid per monomer of CeMIGA2 in the crystal structure (Fig. 1 E), it is not clear where the other lipid binds. A plausible explanation is that it binds in the section of the hydrophobic channel in which we see poorly defined difference density (Fig. 1 E).

### MIGA2 binds lipids and can transfer them between membranes

As MIGA2 is present only in higher eukaryotes, which have different membrane compositions from bacteria, we further analyzed by liquid chromatography/liquid chromatography/MS (LC/LC/MS) lipids that co-purified with hMIGA2_long_ expressed and isolated from mammalian cells (Expi293F). The protein used in these experiments was purified using size exclusion chromatography to remove as much as possible non-specifically bound lipids (Maeda et al., 2013). We found that MIGA2 associated with glycerophospholipids (40 mol % of all lipids bound), fatty acids (FAs, 40 mol % of all lipids bound), and triglycerides (TAG, 10 mol % of all lipids bound). Of the glycerophospholipids, MIGA2 bound mostly phosphatidylcholine (PC) and PE, with PC enriched slightly as compared to its abundance in cells (Fig. 2 B). Phosphatidylserine (PS) and phosphatidylinositol (PI) represented a small fraction of the glycerophospholipids bound. The finding that MIGA2 associates with PC, PE, FA, and TAG is intriguing given the association of MIGA2 with LDs at contact sites, where LDs comprise a neutral lipid core (TAG and cholesterol esters) surrounded by a monolayer of mostly PC and some PE. In cells, TAGs are hydrolyzed to FAs for transport to mitochondria for energy production or to the ER as precursors for glycerol- and glycerophospholipid synthesis. Thus, a possibility is that MIGA2 facilitates fatty acid and glycerophospholipid transfer at mitochondria-LD contacts.

**Figure S2.**
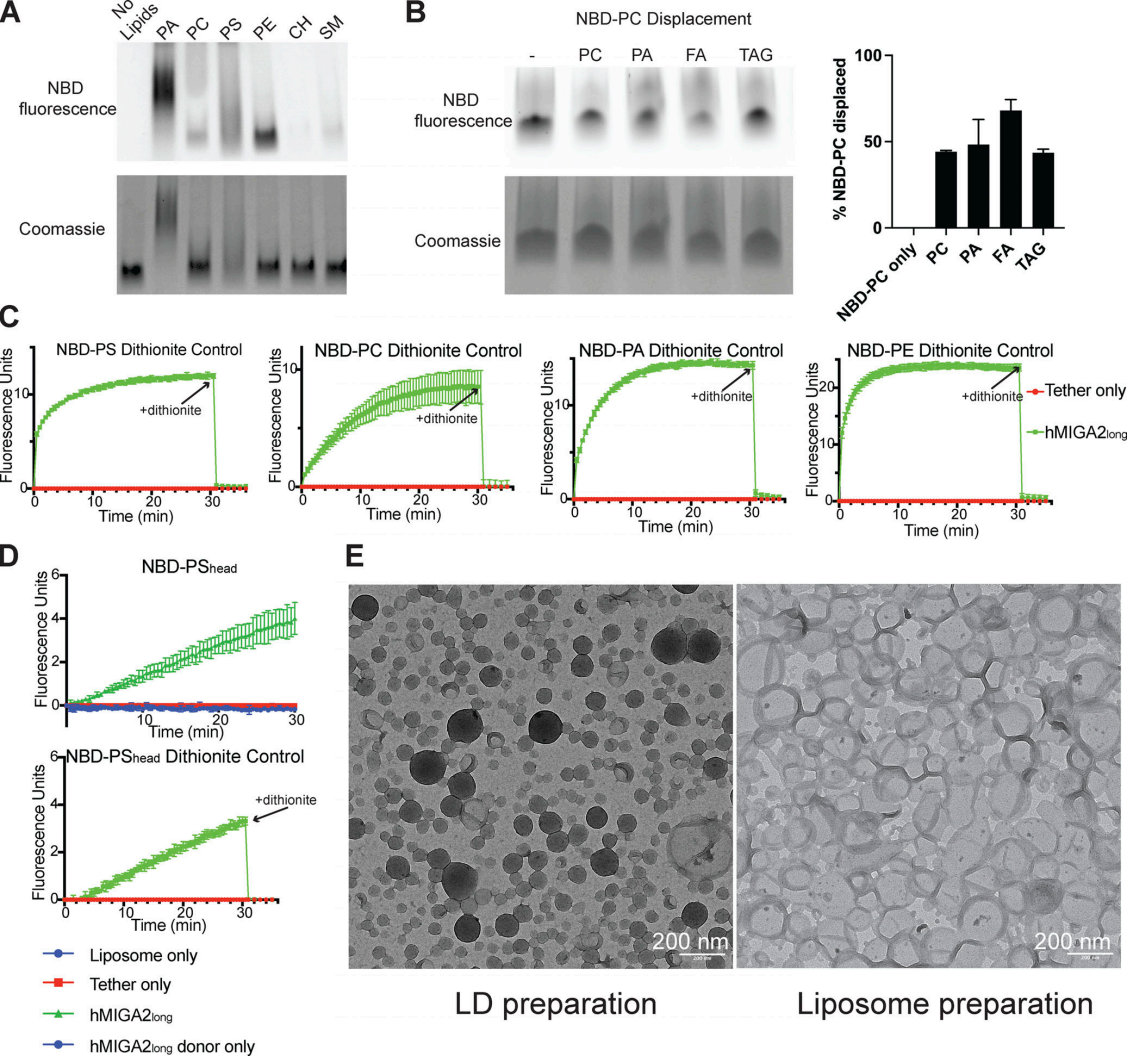
**Lipid binding and transfer by MIGA2. (A)** 3xFLAG-hMIGA2_long_ was incubated with NBD-labeled lipids and examined by native PAGE. Phospholipids (NBD-PA_acyl_, NBD-PC_acyl_ NBD-PS_head_, NBD-PE_acyl_) visualized by their fluorescence, comigrated with protein, visualized by Coomassie blue staining. CH, cholesterol; SM, sphingomyelin. Because migration rates on native gels depend on the mass/charge ratio of the sample, and because MIGA2 dimers can bind multiple lipids at once, MIGA2 incubated with charged lipids migrates as multiple species and is smeared. **(B)** hMIGA2_long_-6xhis was incubated with a 1:1 molar ratio of NBD-PC_acyl_ and unlabeled lipid and examined by native PAGE. **(C)** Dithionite controls for the FRET transfer assay: After the transfer reaction was completed, dithionite was added to rule out the possibility of fusion between donor and acceptor liposomes, which would also result in a fluorescence increase. The fluorescence reduction after dithionite addition is the same for reactions containing hMIGA2 as those without, indicating that fusion has not occurred. Each experiment was performed in triplicate. SDs are shown. **(D)** FRET-based transfer experiment for NBD-PS_head_ and the dithionite control. **(E)** Positive staining TEM micrographs of artificial LDs and liposomes: artificial LDs have a monolayer of phospholipids and TAG core, which are dark under positive staining; liposomes have bilayer membrane of phospholipids and buffer in the core, so that only the membrane is stained dark. Source data are available for this figure: SourceData FS2.

To further assess which lipids MIGA2 might bind, we incubated hMIGA2_long_ with nitrobenzoxadiazole (NBD)-labeled lipids, ran the samples on a native gel, and visualized bound lipids based on their NBD fluorescence. NBD-labeled PC, PS, PE, and phosphatidic acid (PA) are commercially available and co-migrated with hMIGA2_long_; we did not observe significant co-migration with NBD-labeled sterols or sphingomyelin, consistent with the mass spectroscopy analysis described above (Fig. S2 A). The finding that MIGA2 binds PA is of interest as PA plays important roles in mitochondrial biology as a signaling molecule that regulates mitochondrial dynamics and as a precursor for cardiolipin. That PA was not detected among the lipids that co-purified with hMIGA2_long_ (as analyzed by lipidomics, above) reflects that it is present only in minute quantities in cells (van Meer and de Kroon, 2011). An important caveat in interpreting these experiments is that the NBD modification used to visualize the lipids could affect their affinity for MIGA2. However, in a competition experiment, where hMIGA2_long_ was incubated with a 1:1 ratio of NBD-PC and an unmodified lipid, PA displaces half of the NBD-PC, confirming that MIGA2 binds unmodified PA robustly (Fig. S2 B). Similarly, consistent with the lipidomics analysis, MIGA2 can bind FA and TAG as they also displace NBD-PC.

We next assessed whether MIGA2 can transfer lipids between membranes in vitro. To mimic contact sites, we tethered “light” donor liposomes to “heavy” sucrose-filled acceptor liposomes using a previously described linker construct (Bian et al., 2018): it has an N-terminal hexahistidine tag that binds Ni-NTA lipids (5%) in the donor liposome and a C-terminal pleckstrin homolog domain that binds the phosphoinositide PI(4,5)P_2_ (5%) in the acceptor liposomes. Further, a hexahistidine tag was linked to the C-terminus of hMIGA2_long_ or hMIGA2_C_ used in the transfer experiments, allowing robust association with the donor liposomes via the Ni-NTA lipids there. The donor liposomes initially contained either an NBD- or Rhodamine (Rh)-tagged fluorescent lipid species (2%), whereas the acceptor liposomes initially lacked these. hMIGA2 was added to allow for transfer; then, to terminate any transfer reactions (after 30 min), we added protease (proteinase K) to degrade both the tether construct and MIGA2 (Fig. 2 C). The heavy acceptor liposomes were separated from the light donor liposomes by centrifugation, and lipid transfer to the acceptor liposomes was assessed by their Rh or NBD fluorescence. Based on these experiments, MIGA2 (both hMIGA2_long_ and hMIGA2_C_) can bind and robustly transport all four glycerophospholipids, including NBD-PE_acyl_, NBD-PS_acyl_, NBD-PA_acyl_, and, slightly less efficiently, NBD-PC_acyl_, where the NBD label was incorporated into the acyl chain in all cases. (Rh-PE_head_ and NBD-PS_head_, where the fluorescent label is incorporated into the lipid headgroup, were not transferred efficiently, indicating that headgroup modification can interfere with transfer by MIGA2.)

To exclude that the acyl chain modification affects transfer efficiency, we carried out a similar experiment with unmodified PS, using binding by a PS-specific protein (the GST-tagged C2 domain of lactadherin [Moser von Filseck et al., 2015]) rather than fluorescence to quantitate lipid transferred to acceptor liposomes. (In this case, tethering between the donor and acceptor liposomes was disrupted by addition of EDTA and imidazole rather than proteinase K.) The acyl chain modification does not affect transfer by hMIGA2_long_ as PS and NBD-PS were transferred to comparable extents (Fig. 2 C).

In parallel, we used a well-established Förster resonance energy transfer (FRET)–based assay (Kumar et al., 2018; Lees et al., 2017; Saheki et al., 2016) to follow MIGA2-facilitated lipid transfer between membranes (Fig. 2 D). We used a similar tethering strategy as in the end-point assay described above to localize MIGA2 constructs between donor and acceptor liposomes. In this case the donor liposomes initially contain both Rh-PE_head_ (2%) and an NBD-labeled lipid (2%, acyl chain NBD-modified), so that FRET between lipid-associated Rh and NBD quenches the NBD fluorescence. The addition of a lipid transfer protein and consequent transfer of fluorescent lipid species to the acceptor liposomes, which as before initially lack any fluorescent lipid, results in their dilution, decreased FRET between Rh and NBD, and increased NBD fluorescence. As expected, if MIGA2 can transfer fluorescently labeled lipid species, MIGA2 addition led to an increase in fluorescence for all four glycerophospholipids tested (NBD-PE_acyl_, NBD-PS_acyl_, NBD-PA_acyl_, NBD-PC_acyl_). Transfer was slower for NBD-PC_acyl_ but nevertheless robust. (NBD-PS_head_ was transferred, but less efficiently than acyl chain modified NBD-PS [compare Fig. 2 D and Fig. S2 D]).

It has not been established whether lipid transfer proteins might move lipids not only between membrane bilayers but also to/from LDs. Thus, to explore whether MIGA2 might transfer lipids between membranes and LDs, we prepared artificial LDs (Wang et al., 2016) to serve as lipid donors in lieu of donor liposomes in the above FRET-based assay, and assessed NBD-PC_acyl_ or NBD-PE_acyl_ transfer to acceptor liposomes, produced as before. Both lipids are transferred robustly based on increased NBD fluorescence, although PE transfer is more efficient (Fig. 2 E). Our findings lend plausibility to the notion that MIGA2 might transfer lipids—and potentially also FAs—between mitochondria and LDs in vivo. (An experiment to assess whether FAs enzymatically produced from LD TAGs can be transferred into other organelles is beyond the scope of this work.)

Thus, the contact site protein MIGA2 can bind glycerophospholipids and FAs within a hydrophobic channel and robustly transfers glycerophospholipids and possibly also FAs, although this remains to be tested, between membranes in vitro. As this manuscript was in preparation, Kim et al. (2022) reported the structure of MIGA2_C_ from zebrafish (the structure is nearly identical to the AlphaFold2 prediction as well as CeMIGA2_C_; the RMSD between experimental structures is 1.2 Å over 212 Cα’s) and used FRET-based assays similar to ours, with fluorescently modified lipids, to show that it transfers glycerophospholipids in vitro. They observed robust lipid transfer only in the case of NBD-PS_acyl_, not NBD-PA_acyl_, -PC_acyl_, or headgroup modified NBD-PE_head_, and suggested that MIGA2 preferentially transfers PS. Their experimental setup differs from ours in several aspects. Most significantly, even while they used only a short construct corresponding to MIGA2_C_ (which may lack modules required for membrane association), they did not use a tethering strategy in their assays. Tethering MIGA2 to the liposomes assures that the protein interacts with the liposome, providing an opportunity for it to capture/extract lipid substrate, a stochastic event that determines the rate of the lipid transfer reaction. In the absence of a tether, whether a protein stays associated with membrane long enough for efficient lipid extraction depends heavily on the donor membrane composition (for example, see Horenkamp et al., 2018; Zhang et al., 2022); often the presence of acidic lipid (Kim et al. [2022] used 10% NBD-PS, much more than would be present in ER, mitochondria, or LDs) enhances protein interaction with membranes. We suspect that the transfer rates they observed reflect differences in lipid extraction rates arising from different donor liposome compositions and membrane characteristics, and that transfer of NBD-PC, -PA, and -PE would be enhanced upon addition of unlabeled PS to the donor liposomes. They also compared transfer of NBD-PS, harboring the NBD modification in the acyl chain, with NBD-PE_head_, where NBD was incorporated into the headgroup, and attributed the difference to a preference for the PS headgroup. As shown in Fig. 2, C and D, and Fig. S2 D, however, headgroup modifications can interfere with MIGA2-mediated transport, whereas the acyl chain modification does not. In contrast, consistently using acyl-chain modified NBD-lipids, we observe robust transfer of all four glycerophospholipids tested, irrespective of headgroup. Further, arguing that PS is not a selective cargo, PS is not enriched in the pool of glycerophospholipids that co-purifies with MIGA2 in our lipidomics analysis. This is in contrast to other PS-specific transfer proteins like Osh6 (Maeda et al., 2013). Thus, MIGA2 most likely transfers not only PS but a variety of glycerophospholipids in vivo.

### Its lipid transfer ability is essential for MIGA2 function in vivo

**Figure 3.**
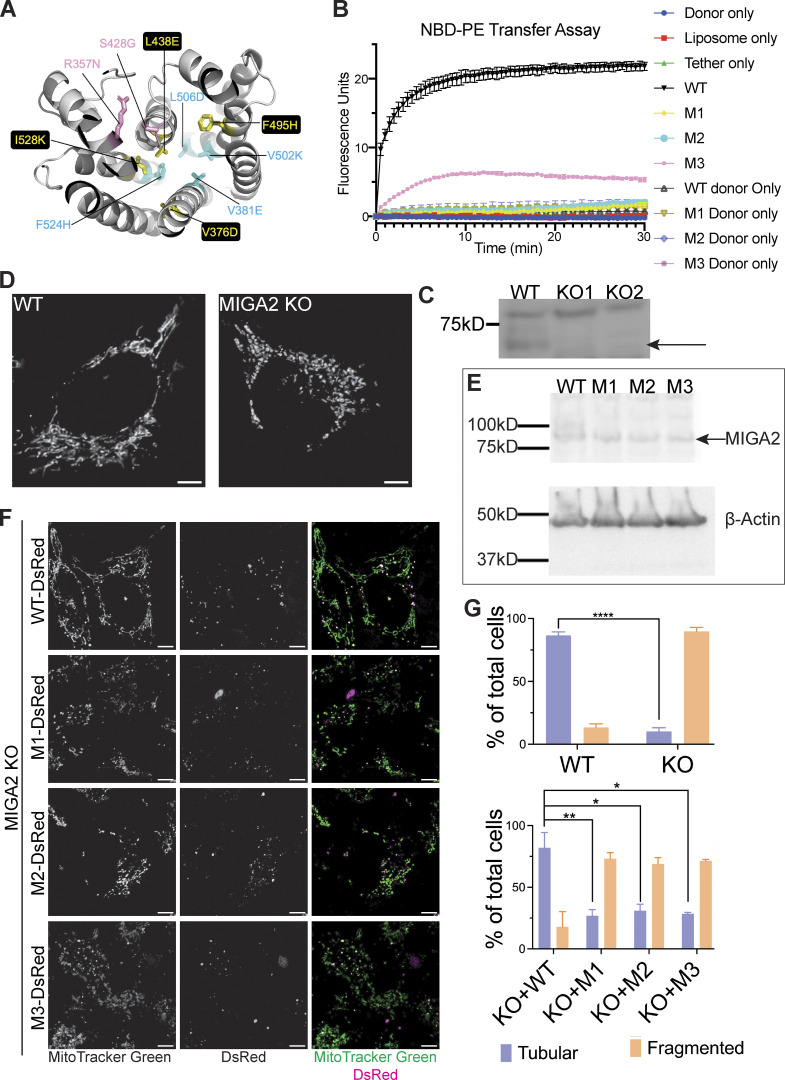
**MIGA2’s lipid transfer ability is essential for its function in mitochondria. (A)** In two mutant forms of hMIGA2 (M1 and M2), hydrophobic residues in the lipid binding channel were altered to hydrophilic amino acids, indicated in yellow and cyan, respectively. In a third mutant (M3), in pink, a strictly conserved residue near the lipid headgroup was changed to asparagine (R357N in hMIGA2). The hMIGA2_C_ shown was modeled in AlphaFold2. **(B)** All three mutants lose ability to transfer lipids in the FRET-based assay with NBD-PE_acyl_. Each experiment was performed in triplicate. SDs are shown. **(C)** Knockout of MIGA2 in Hela cells was confirmed by Western blot against MIGA2. The upper non-specific band served as an internal loading control. The arrow indicates the band of MIGA2. **(D)** Mitochondria stained with MitoTracker Green in WT Hela cells are tubular, while in MIGA2 KO cells are fragmented. **(E)** Expression of WT MIGA2-DsRed and M1-, M2-, and M3-DsRed mutants in MIGA2 KO cells was compared by Western blot against MIGA2. The four MIGA2 constructs expressed at the same level when normalized to β-actin. **(F)** Expression of WT MIGA2-DsRed (PGK) in the KO cells rescued the mitochondria fragmentation morphology, whereas cells expressing either M1-, M2-, or M3-DsRed (PGK), the lipid transfer incompetent constructs, showed fragmented mitochondria. The dot expression pattern of MIGA2 constructs was consistent with previous studies (Zhang et al., 2016). **(G)** Quantification of tubular and fragmented mitochondria in different cells (*n* > 50). Statistical significance was calculated by Welch’s two-tailed unpaired *t* test. Results were indicated in the following manner: ∗ for P < 0.05, ∗∗ for P < 0.01, ∗∗∗∗ for P < 0.0001, where P < 0.05 is considered as significantly different. Graphs in the same panel are displayed with the same brightness and contrast settings. Scale bars in D and F, 5 μm. Source data are available for this figure: SourceData F3.

Like many other lipid transport proteins, MIGA2 was first identified as a tether that promotes contact site formation. As a first step in determining whether, in addition to its tethering ability, MIGA2’s lipid transfer activity might be biologically important, we designed lipid transport incompetent mutants for use in functional studies. We introduced clusters of hydrophilic residues into the lipid binding channel of human MIGA2 so as to interfere with lipid solubilization (M1: V376D/L438E/F495H/I528K; M2: V381E/V502K/L506D/F524H; Fig. 3 A). In another construct, we mutated the strictly conserved arginine near the lipid headgroup in the crystal structure (M3: R357N/S428G, corresponding to R177/S248 in CeMIGA2, respectively). The mutations did not interfere with protein folding as assessed by size exclusion chromatography, where the mutant and WT proteins migrated in the same way (Fig. S1 C), nor did the mutations interfere with MIGA2 localization in cells (Fig. S1 D). In vitro, in the FRET-based assay described above with NBD-PE_acyl_, all three mutant constructs (M1, M2, M3) are lipid transfer impaired (Fig. 3 B).

Zhang et al. (2016) showed that mitochondria are fragmented in MIGA2 knockout (KO) cells. As a tool to assess whether a lack of MIGA2 lipid transport ability might be responsible for this mitochondrial phenotype, we used CRISPR/Cas9 technology to make a MIGA2 KO Hela cell line (Fig. 3 C). We stained mitochondria with MitoTracker Green in WT cells, the KO cells, and cells transiently expressing WT MIGA2-DsRed or mutated versions (M1-DsRed, M2-DsRed, or M3-DsRed), then imaged them. To avoid mitochondria clustering caused by MIGA2 overexpression (Zhang et al., 2016), we used a relatively weak promotor, PGK, for exogenous MIGA2-DsRed expression. 90% of MIGA2 KO cells had fragmented, dot-like mitochondria, significantly different from WT cells, which mostly had tubular mitochondria (∼90%; Fig. 3, D and G). More importantly, transiently expressing WT MIGA2-DsRed in the KO cells rescued mitochondrial morphology in 80% of the cells, whereas only ∼30% of the cells expressing the lipid transfer incompetent constructs (M1-DsRed, M2-DsRed, or M3-DsRed) had tubular mitochondria (Fig. 3, F and G). The expression levels for the transiently expressed MIGA2 WT and mutants were the same (Fig. 3 E). This indicates that the lipid transport ability of MIGA2 is necessary for its function in mitochondria.

**Figure 4.**
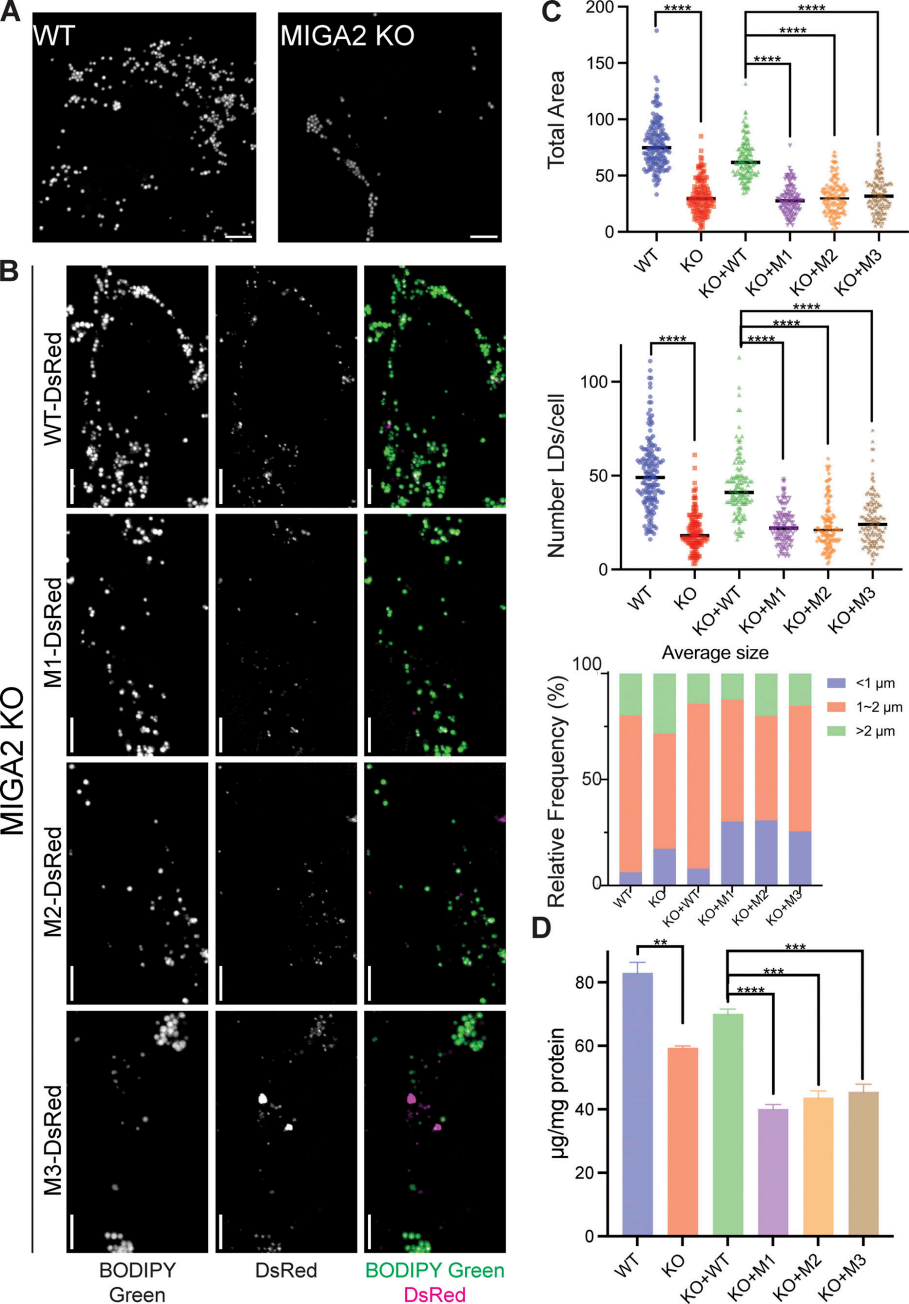
**MIGA2’s lipid transfer ability is essential for its function in LD biology. (A)** LDs were stained with BODIPY green 493/503 and imaged in cells treated with 100 μM OA for 24 h. MIGA2 KO Hela cells had fewer and more small LDs than the WT cells. Scale bar, 5 μm. **(B)** The phenotype of fewer and smaller LDs in MIGA2 KO cells was reversed in those cells expressing MIGA2-DsRed (PGK promoter). In contrast, MIGA2 mutants (PGK) failed to do so, including when they were expressed at higher levels (Fig. S3, C and D). Scale bar, 5 μm. **(C)** Quantification of B: LD total area and number per cell and average size frequency in different cells. **(D)** TAG content in different cells in WT and MIGA2 KO cells. Statistical significance was calculated by Welch’s two-tailed unpaired *t* test. Results were indicated in the following manner: ∗∗ for P < 0.01, ∗∗∗ for P < 0.001, ∗∗∗∗ for P < 0.0001, where P < 0.05 is considered as significantly different.

**Figure S3.**
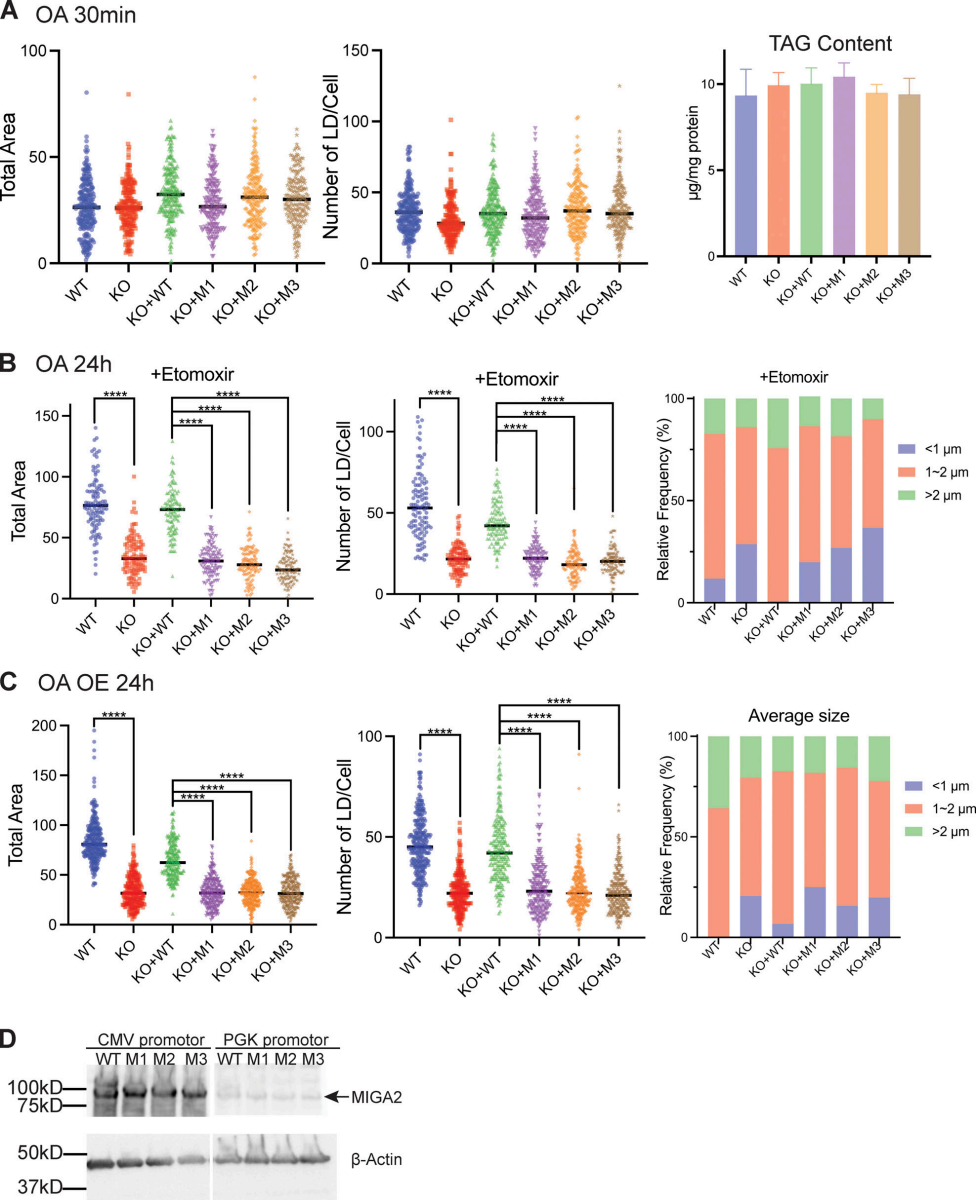
**Cellular LD and TAG contents. (A)** Left and middle: LD quantification of cells with or without transfection of MIGA2-DsRed (CMV) and treated with OA for 30 min. Right: TAG content of the cells with same treatment. There is no significant difference between WT and KO cells, or between rescue by WT and mutant MIGA2-DsRed constructs in terms of LD and TAG abundance. **(B)** LD quantification of cells with or without transfection of MIGA2-DsRed (CMV) and treated with etomoxir for 2 h and OA for 24 h. **** for P < 0.0001, where P < 0.05 is considered significantly different. **(C)** LD quantification of cells with or without transfection of MIGA2-DsRed constructs (CMV, expression level is at 4–5 times higher as compared with PGK promotor) and treated with OA for 24 h. This showed similar pattern with cells transfected with PGK MIGA2-DsRed (Fig. 4 C). **** for P < 0.0001, where P < 0.05 is considered significantly different. **(D)** Expression levels for MIGA2-DsRed constructs under the CMV versus PGK promoter, as in Fig. 3 E. Source data are available for this figure: SourceData FS3.

Freyre et al. (2019) discovered that MIGA2 targets to LDs and participates in their formation. Using 24 h oleic acid treatment to induce LD formation in Hela cells, we likewise found that depletion of MIGA2 in the KO cells reduced the number and size of LDs and total cellular TAG content (Fig. 4). We excluded that this phenotype is due to impaired TAG synthesis in the KO cells (cellular TAG levels in the WT and MIGA2 KO cells following short [30 min] oleate treatment are the same; Fig. S3 A) or that the difference is due to changes in fatty acid oxidation (LD size and number in the WT versus KO cells were not affected when the cells were treated with etomoxir to block mitochondrial fatty acid oxidation, Fig. S3 B). Moreover, we found that the phenotype of fewer and smaller LDs and less TAG in MIGA2 KO cells was reversed in cells expressing a WT construct, MIGA2-DsRed (Fig. 4, B–D), but not in cells expressing lipid transport impaired mutants (M1-DsRed, M2-DsRed, or M3-DsRed), regardless of their expression level (Fig. 4, B–D; and Fig. S3, C and D). Thus, the lipid transfer ability of MIGA2 is required for LD generation and growth. A plausible role for MIGA2 is to supply phospholipids for LD monolayer expansion.

### Possible mechanisms for MIGA2 function

The lipid transport ability of MIGA2 is thus intrinsic to its functions both in mitochondrial and LD biology. Mitochondria rely on protein-mediated lipid transport at contact sites, especially with the ER, for most of their membrane lipids as they are disconnected from vesicle trafficking pathways. A number of lipid transport proteins have already been identified at ER-mitochondrial contacts, and it will be important to discover a distinguishing role for MIGA2 at these sites. Perhaps it supplies mitochondria with PA, or perhaps as proposed by Kim et al. (2022) it participates in PS transfer to the mitochondria, where PS is a precursor for PE; or it might exchange PE and PS, transferring PE from mitochondria back to the ER. Or MIGA2 could act as a non-specific glycerophospholipid transporter that equilibrates the lipid compositions of ER and mitochondrial membranes at contact sites. LD-mitochondrial contacts have been implicated in LD biogenesis previously (Benador et al., 2018). That lipid exchange takes place at these sites is not well established (but see Du et al. [2020]). It is nevertheless possible that MIGA2-mediated lipid transport there plays a role in LD formation; or maybe MIGA2 acts at three-way contacts between the LDs, mitochondria, and the ER, where de novo LD formation takes place (reviewed in Choudhary and Schneiter, 2021; Henne et al., 2020; Olzmann and Carvalho, 2019). To us, a very interesting possibility, given that MIGA2 co-purifies with, binds, and transfers PC and PE (the prominent glycerophospholipids in the monolayer surrounding the LD core), and binds FAs (produced from TAGs in the LD core), is that it may facilitate PC/PE and FA transport between LDs and mitochondria at LD-mitochondrial contacts and FA transport on to other organelles from there, to play a role in ATP generation (mitochondria) or else as a precursor for glycerolipid synthesis (ER). To our knowledge, no FA transfer protein has yet been identified. Further studies that identify the physiological ligands of MIGA2 are a next step in understanding the molecular basis of MIGA2 function.

## Materials and methods

### Materials

Rabbit polyclonal anti MIGA2 antibody (ab 122713) was purchased from Abcam. Rabbit monoclonal anti β-actin antibody was from Cell Signaling Technology (4970; RRID:AB 2223172). Almost all lipids were purchased from Avanti Polar Lipids: DOPC (850375), liver PE (840026), DGS-NTA (Ni; 709404), Liss Rhod PE (810150), Brain PI (4,5) P2 (840046), NBD-PA (810176), NBD-PS (810195), 18:1 NBD-PS (810198), NBD-PC (810133), NBD-PE (810156), NBD-cholesterol (810250), NBD-sphingomylein (810219), 16:0 PA (830855), triolein (18:1 TG; 870110). Palmitic acid (P0500) was ordered from Sigma-Aldrich. Sodium dithionite was from Sigma-Aldrich (157953). Etomoxir was purchased from MedChemExpress (HY-50202). The Hela cell line (ATCC #CCL-2; RRID: CVCL 0030) was a gift from Mals Mariappan (Yale University, New Haven, CT), *C. elegans* cDNA was a gift from Daniel Colon-Ramos (Yale University, New Haven, CT; Xuan et al., 2017). Plasmid for the 6xhis-PH-tethering construct (Bian et al., 2018) was a gift from Pietro De Camilli (Yale University, New Haven, CT).

### Plasmid construction

*C. elegans* MIGA (Uniprot Q21096) fragments were amplified from *C. elegans* cDNA library (Xuan et al., 2017)and subcloned into pET28-6xhis-sumo plasmid (V012798; NoVoPro) or pET29 (69871; Addgene) or pCMV10 plasmid (E7658; Sigma-Aldrich) with C-terminal 6xhis tag or N-terminal 3xFLAG tag. Human MIGA2 (Uniprot Q7L4E1) gene was codon-optimized for bacterial expression and synthesized by Genescript. The gene fragments (170–593, 306–593) were PCR amplified and cloned into pCMV10 or pET29 plasmid with N-terminal 3xFLAG or a C-terminal 6xhis tag. The mutant gene fragments were synthesized by Genescript and PCR amplified and cloned into either pCMV10 plasmid with a N-terminal 3xFLAG and a C-terminal 6xhis tag for biochemical experiments, or pLENTi-PGK plasmid (Valverde et al., 2019) or pLVX-CMV plasmid (632159; Clonetech) with a C-terminal DsRed tag for imaging and cellular experiments. Primers used in this study are in Table S1.

### Protein expression and purification

#### For crystallization

The pET28-6xhis-sumo-CeMIGA (106–496) construct was expressed in C43 (DE3) *E. coli* cells (CMC0019; Sigma Aldrich). Cells were grown at 37°C to an OD_600_ of 0.6–0.8, when protein expression was induced with 0.8 mM IPTG, and then cells were cultured at 18°C for another 6 h. Cells were pelleted, resuspended in buffer A (20 mM Hepes, pH 7.4, 200 mM NaCl, 1 mM tris(2-carboxyethyl)phosphine (TCEP), and 5% glycerol) containing 1× complete EDTA-free protease inhibitor cocktail (1187358001; Roche) and lysed in an Emulsiflex-C5 cell disruptor (Avestin). Cell lysates were clarified via centrifugation at 27,000 *g* for 30 min. To collect the protein, supernatant was incubated with Ni-NTA resin (30210; Qiagen) for 1 h at 4°C, and then the resin was washed tandemly with buffer B (buffer A + 0.005% Triton X-100), buffer C (buffer B + 10 mM imidazole), buffer D (buffer B + 20 mM imidazole), buffer E (buffer B + 40 mM imidazole), buffer A to remove extra Triton X-100. Retained protein was eluted from the resin with buffer A supplemented with 250 mM imidazole. Sumo protease was added to digest overnight at 4°C. After removing imidazole by buffer exchange, the sample was bound to the Ni-NTA resin again to remove protease and 6xhis-sumo. The flow-through was collected, concentrated in a 30-kD molecular weight cutoff Amicon centrifugal filtration device, and loaded onto a Superdex 200 16/60 column (GE Healthcare) equilibrated with buffer F (20 mM Hepes, pH 7.4, 200 mM NaCl, 1 mM TCEP). Peak fractions containing pure MIGA were recovered and concentrated.

#### For biochemical assays

3xFLAG-MIGA2_long_ WT and mutants for Expi293 cell expression were transfected into Expi293F cells (A14527; Thermo Fisher Scientific) according to manufacturer instructions for 48 h. Cells were pelleted and resuspended in buffer A and lysed by five freeze–thaw cycles. Cell lysates were clarified via centrifugation at 27,000 *g* for 30 min, and the supernatant was incubated with preequilibrated anti-FLAG M2 affinity resin (A2220; Sigma-Aldrich) for 2 h at 4°C. The resin was washed with buffer A and incubated overnight with buffer A containing 2.5 mM ATP and 5 mM MgCl_2_. The protein was eluted with buffer A supplemented with 0.2 mg/ml 3× FLAG peptide (A6001; APExBio), concentrated in a 30-kD molecular weight cutoff Amicon centrifugal filtration device, and loaded onto a Superdex 200 10/30 column (GE Healthcare) equilibrated with buffer A. Peak fractions were pooled and concentrated.

MIGA2_long_-6xhis and MIGA2_C_-6xhis were expressed in BL21 (DE3) *E. coli* cells (69450; Sigma-Aldrich). The expression and purification of these constructs were the same as that for CeMIGA(106–496) except that no detergent added during washes, and no sumo digestion and rebinding, instead loading onto Superdex 200 16/60 column directly after elution and concentration.

The 6xhis-PH-tether construct was purified as described before (Bian et al., 2018).

The PS-specific probe (GST-C2_Lact_) was purified as described before (Horenkamp et al., 2018; Moser von Filseck et al., 2015).

### Protein crystallization, structure determination, and refinement

Crystals of *C. elegans* MIGA (106–496) at 6 mg/ml were grown at 18°C using the sitting-drop vapor-diffusion method. Equal volumes of protein and reservoir solution (0.2 M sodium malonate, pH 7.4, 22% PEG3350) were mixed. Crystals, which belonged to spacegroup P3_1_21 (a = 91.3, b = 91.3, c = 366.74 Å), were transferred to solutions that also included cryo-protectants, and flash frozen in liquid nitrogen. We examined ∼100 crystals. Although most did not diffract to atomic resolution, a small number diffracted to ∼4 Å or better. We found a single crystal among them cryo-protected in 25% ethylene glycol that diffracted to 3.3 Å, from which we collected the data set used in structure determination. Diffraction data were collected at the Northeastern Collaborative Access Team (NE-CAT) beamline 24-ID-C at the Advanced Photon Source, using a Dectris EIGER2 X 16M pixel array detector, and processed using XDS (Kabsch, 2010; RRID:SCR_015652; http://xds.mpimf-heidelberg.mpg.de). Statistics for data collection are shown in Table 1. For phasing, we used molecular replacement with Phaser MR (McCoy et al., 2007; RRID:SCR_014219; https://www.phenix-online.org) using a CeMIGA2_C_ model generated by AlphaFold2 Colab (Tunyasuvunakool et al., 2021; https://colab.research.google.com/github/deepmind/alphafold/blob/main/notebooks/AlphaFold.ipynb) preprocessed with phenix.process_predicted_model (Liebschner et al., 2019) to remove the low-confidence loop regions. Four copies of CeMIGA2_C_ were placed in the asymmetric unit. Initial maps showed clear density for two four-helix bundles, representing dimers of the coiled-coil helices from the CeMIGA2 linker. The helices were modeled manually in Coot (Emsley et al., 2010; RRID:SCR_014222; https://www2.mrc-lmb.cam.ac.uk/personal/pemsley/coot/). Additional unmodeled density was observed in the Fo-Fc maps within the hydrophobic cavity in two MIGA2_C_ domains, which clearly corresponded to bound glycerophospholipids. We built PE 16:0 (chemical id PEF) into these densities and eventually, using non-crystallographic symmetry, placed PE into less-well-defined densities in the remaining two monomers. The refinement consisted of cycles of manual rebuilding in Coot and automated refinement in Phenix (Liebschner et al., 2019), including isotropic b-factors, Translation-Libration-Screw refinement, and non-crystallographic symmetry restraints. Refinement statistics are in Table 1. The coordinates and structure factors have been deposited in the PDB (accession no. 8EDV).

**Table 1.** Data collection and refinement statistics

	CeMIGA2_long_
Wavelength (Å)	0.979110
Space group	P 3_1_ 2 1
Cell dimensions	
a, b, c (Å)	91.3, 91.3, 366.7
α, β, γ (°)	90, 90, 120
Monomers/asymmetric unit	4
Data collection	
Resolution range (Å)	48.36–3.30 (3.42–3.30)
Completeness (%)	99.9 (99.2)
Redundancy	10.2 (10.5)
I/σ(I)	14 (1.6)
Rmerge (%)	16.9 (73.6)
Rmeas (%)	17.9 (77.4)
Rpim (%)	5.6 (23.6)
CC (1/2)	99.3 (83.5)
Refinement	
Rwork (%)	26.98
Rfree (%)	29.56
RMSD bond angles/lengths	0.515/0.002
Ramachandran statistics (%)	95.38/3.93/0.69
PDB accession code	8EDV

Values in parentheses correspond to the highest resolution shell. The Ramachandran statistics show the percentage of residues in favored/allowed/other regions.

Figures were made using Pymol (The PyMOL Molecular Graphics System, Version 1.5.0.4. Schrödinger, LLC; RRID:SCR_000305; https://www.pymol.org).

### Lipid co-migration assay

Purified hMIGA2_long_ was mixed with 1 μl of NBD-labeled lipids (at 1 mg/ml) in 20 μl total reaction volumes and incubated on ice for 2 h. Samples were loaded onto 4–15% Mini-Protean Precast Native gels and run for 90 min at 100 V. NBD fluorescence was visualized using an ImageQuant LAS4000 (GE Healthcare; RRID:SCR_014246; http://gelifesciences.com/webapp/wcs/stores/servlet/catalog/en/GELifeSciences-us/products/AlternativeProductStructure_16016/29000605). Then gels were stained with Coomassie blue G250 to visualize total protein. Images were analyzed via Fiji (ImageJ; RRID:SCR_002285; https://fiji.sc).

### Lipid competition assay

NBD–PC and other non-labeled lipids at a 1:1 molar ratio (1.13 mM each) were mixed with hMIGA2_long_ in 40 μl reaction volumes and incubated on ice for 2 h. Samples were loaded onto 4–15% Mini-Protean Precast Native gels and run for 90 min at 100 V. NBD fluorescence was visualized using an ImageQuant LAS4000 (GE Healthcare).

### Liposome preparation

#### For FRET-based assay

Lipids in chloroform were mixed (donor liposomes: 61% 1,2-Dioleoyl-sn-glycero-3-phosphocholine [DOPC], 30% liver PE, 2% NBD-labeled lipids [all NBD-labeled lipids are 18:1–12:0 with NBD incorporated into the fatty acyl chain, except for NBD-PS_head_], 2% Rh-PE, and 5% DGS-NTA (Ni); acceptor liposomes: 65% DOPC, 30% liver PE, and 5% PI[4,5]P_2_) and dried to thin films and vacuumed for 30 min. Lipids were subsequently dissolved in buffer F at a total lipid concentration of 1 mM and incubated at 37°C for 1 h, vortexing every 10–15 min. Liposomes were subjected to 10 freeze–thaw cycles alternating between liquid nitrogen and room temperature water bath with vortexing every three cycles. Crude liposomes were then extruded through a polycarbonate filter with 100 nm pore size a total of 11 times via a mini extruder (Avanti Polar Lipids) and used within 24 h.

#### For end-point transfer assay

Light donor liposomes (63% DOPC, 30% liver PE, 2% fluorescently labeled lipids, and 5% DGS-NTA [Ni]) were prepared similarly as above except that either NBD-lipids or Rh-PE, but not both, were included. The donor liposomes for the C2_Lact_-PS-Probe based assay were composed of 60% DOPC, 30% liver PE, 5% PS, and 5% DGS-NTA (Ni). For heavy acceptor liposomes, lipids in chloroform were mixed, dried, and vacuumed as above. The lipid film was dissolved in buffer F containing 0.75 M sucrose at a total lipid concentration of 1 mM and incubated at 37°C for 1 h, vortexing every 10–15 min. Liposomes were subjected to 10 freeze–thaw cycles, and then mixed with 2 volumes of buffer F and pelleted for 40 min at 18,500 *g*. The resulting liposome pellet was resuspended in buffer F and pelleted again for 30 min. The second pellet was resuspended to 1 mM total lipid concentration in buffer F and used immediately.

### Artificial LD preparation

Artificial LDs were prepared according to Wang et al. (2016). Briefly, 2 mg of total phospholipids (81% DOPC, 10% 1,2-dioleoyl-sn-glycero-3-phosphoethanolamine, 2% NBD-labeled lipids, 2% Rh-PE, and 5% DGS-NTA [Ni] by molar ratio) were mixed and dried to thin films and vacuumed for 30 min. 5 mg of TAG (T7140; Sigma-Aldrich) was added to the phospholipid film. The lipids were resuspended in 100 μl buffer F. The sample was vortexed for 4 min, with 10 s on, 10 s off. The milky lipid mixture was centrifuged at 20,000 *g* for 5 min. The fraction containing artificial LDs formed a floating white band at the top of the tube. The underlying solution and pellet were removed. This process was repeated until no pellet formed upon centrifugation. The white band was resuspended in 100 μl buffer F, and centrifuged at 1000 *g* for 5 min. The solution underneath the floating white band was collected. The low-speed centrifuge was repeated until there is no white band formation after centrifuge. The concentration of lipids in the final solution was determined by NBD fluorescence compared with the liposomes containing the same ratio of NBD- and Rh-lipids. The LDs were stable at 4°C for a week.

### Positive staining of artificial LDs or liposomes

The positive staining was done as described before (Wang et al., 2016), Briefly, 8 μl of purified artificial LDs or liposomes were loaded onto the glow-discharged carbon film coated grids (CF400-CU; Electron Microscopy Sciences) for 1 min followed by blotting with filter paper to remove extra sample. The sample was then fixed with 1% osmium tetroxide for 10 min and washed three times with deionized water. The sample was stained with 0.1% tannic acid for 5 min and 2% uranyl acetate for 5 min and washed with deionized water. The micrographs were recorded on a Talos microscope at 110 kV with 36,000 magnification at Yale Science Hill EM facility.

### In vitro lipid transfer assay

#### FRET-based assay

Lipid transfer experiments were set up at 30°C in 96-well plates, with 100 μl reaction volumes containing 200 µM lipids in donor liposomes and 200 μM lipids in acceptor liposomes. Proteins (0.25 µM each of tether and lipid transport protein) were added to start the reaction, and after excitation at 460 nm, NBD emission (538 nm) was monitored for 30 min using a Synergy H1 Multi-Mode Microplate Reader (Agilent). For transfer from artificial LDs to liposomes, 80 μM lipids in donor LDs and 80 μM lipids in acceptor liposomes, and 0.25 μM proteins were used.

#### Dithionite assay

Lipid transfer assays were performed as above, except for the addition of freshly prepared dithionite (to 5 mM final concentration) after the last time point, and NBD fluorescence was monitored for an additional 5 min.

#### End-point transfer assay

Lipid transfer reactions were performed in 100 μl volumes. The reaction was initiated by adding 250 µM lipids each in light donor and in heavy acceptor liposomes into 0.32 μM protein (tether and hMIGA2_long_ or hMIGA2_C_) and terminated by the addition of 10 μl terminate cocktail (2 mg/ml proteinase K, 1.8 M imidazole, 50 mM EDTA). The complete digestion of the proteins was confirmed by SDS-PAGE gel. Light and heavy liposomes were then separated by centrifugation at 18,500 *g* for 15 min, and heavy liposomes recovered in the pellets were resuspended in 100 μl buffer F. Fluorescence signal (NBD: ex 440 nm, em 514 nm; Rh: ex 550 nm, em 590 nm) present in the pellet was determined using a Synergy H1 Multi-Mode Microplate Reader (Agilent). A separate standard curve of fluorescence intensity to the amount of fluorescent lipids was made for NBD-PS (both acyl or headgroup modified) and Rh-PE, and the amount of fluorescent lipids being transferred in the assay was determined by the standard curve. For the C2lactadherin PS-probe based transfer assay, after the reaction, 10× terminating cocktail (1.8 M imidazole, 100 mM EDTA) was added before the donor and acceptor liposomes were separated. Donor and acceptor fractions were analyzed by SDS-PAGE gel to quantify the PS probe in each of the fractions.

### MS analysis of hMIGA2_long_

#### Lipidomic analysis

Gel filtrated 3xFLAG-hMIGA2_long_ was sent to Michigan State University’s Collaborative Mass Spectrometry Core for untargeted lipidomics analysis. The sample was spiked with internal standards and calibration mixture. Lipids were extracted with methyl tert-butyl ether twice, and after drying, resuspended in isopropanol containing 0.01% butylated hydroxy toluene. The sample was resolved by Shimadzu Prominance high performance LC and lipid species were detected by a Thermo Fisher Scientific LTQ-Orbitrap Velos mass spectrometer in both positive and negative ionization modes. Lipid species were quantified based on internal standards and summed by lipid class.

#### Native MS

The hMIGA2 sample was buffer exchanged to 200 mM ammonium acetate (MP Biomedicals), 2 mM DTT with Zeba Spin Desalting Columns (Thermo Fisher Scientific). The protein concentration in the analyzed sample was in the range between 1 and 5 μM. Native MS was performed on Q Exactive UHMR (Thermo Fisher Scientific) using in-house nano-emitter capillaries. The tips in the capillaries were formed by pulling borosilicate glass capillaries (OD: 1.2 mm, ID: 0.69 mm, length: 10 cm, Sutter Instruments) using a Flaming/Brown micropipette puller (ModelP-1000, Sutter Instruments). Then the nano-emitters were coated with gold using rotary pumped coater Q150R Plus (Quorum Technologies). To perform the measurement the emitter filled with the sample was installed into Nanospray Flex Ion Source (Thermo Fisher Scientific). MS parameters for the analysis of the proteins or protein complexes include spray voltage 1.1–1.3 kV, capillary temperature 275°C, resolving power 6,250 at m/z of 400, ultrahigh vacuum pressure 4.6e−10–8.18e−10, in-source trapping between −100 V and −200 V.

### Functional experiments

#### Cell culture and transfection

Hela cells were cultured in DMEM (11965092; Thermo Fisher Scientific) supplemented with 10% FBS (10438062; Thermo Fisher Scientific) and 1% penicillin-streptomycin (15140122; Thermo Fisher Scientific) at 37°C in a 5% CO_2_ incubator. Cells were used in experiments before passage 5. DNA transfection was performed using Lipofectamine 3000 (L3000015; Thermo Fisher Scientific) according to the manufacturer’s instruction.

#### Generation of MIGA2 KO cell line

CRISPR gRNAs targeting the third exons of MIGA2 were designed using the online TKO CRISPR Design Tool (https://crispr.ccbr.utoronto.ca/crisprdb/public/library/TKOv3/). 5′-GCG​GAA​AGT​CCT​CTT​TGC​CA-3′ was chosen as gRNA to knock out MIGA2. The gRNA was cloned into pX458 (48138; Addgene) as described previously (Ran et al., 2013). Hela cells were transiently transfected with the constructs containing gRNAs. After 48 h, the GFP positive individual cells were selected by flow cytometry (BD FACSAria) and seeded in 96-well plates for single clones. The clones were validated by genotyping and Western blotting. For genotyping, briefly, genomic DNAs from single clones were extracted using QuickExtract DNA extraction solution (QE0905T; Lucigen), and PCR products containing the site of Cas9 cleavage site were generated using the following primers: 5′-TAG​ACC​TCA​CCT​TCT​CGG​CAC​T-3′; 5′-CCA​ATA​TCC​CCA​AGT​AGA​GAG​TG-3′. PCR products were sequenced.

#### Western blotting

The samples were denatured by the addition of SDS sample buffer (125 mM Tris-HCl, pH 6.8, 16.7% glycerol, 3% SDS, 0.042% bromophenol blue) and heated at 95°C for 5 min. The proteins were separated using denaturing SDS-PAGE and analyzed by immunoblotting. The proteins were transferred onto a polyvinylidene fluoride membrane. After the transfer the membranes were incubated in 5% nonfat dry-milk–TBST (20 mM Tris-HCl, pH 7.6; 150 mM NaCl; 0.1% Tween 20) for 1 h at room temperature. The membranes were then washed three times and incubated with primary antibodies at 1:1,000 dilution in 5% BSA-TBST over night at 4°C or at room temperature for 90 min. The membranes were washed three times and incubated for 60 min with secondary antibodies coupled to horse-radish peroxidase in TBST containing 5% nonfat dry-milk. The proteins of interest were developed by ECL (BioRad) and visualized using ImageQuant LAS4000 (GE Healthcare).

#### Lipid extraction and TAG measurement

Cells with or without transfection of the MIGA2-DsRed (Cytomegalovirus promoter, CMV) constructs in 10 cm dishes were treated with oleic acid (OA) for indicated times. Cells were washed twice with PBS and collected in PBS with cell scrapers; 1/10 of the sample was set aside for protein quantification. Protein amount was determined by Bradford (Bio-Rad) assay at 595 nm absorbance. Lipid extraction was done as in Du and Yang (2020). Briefly, the cell pellet was resuspended in hexane-isopropanol (3:2) solvent, then agitated at room temperature for 30 min to extract lipids. The organic solvent was transferred into glass tube and dried overnight in a chemical hood. The lipid film was resuspended in 200 μl methanol-chloroform (2:1) and vortexed. The samples and a series of TAG standard dilutions were mixed with enzyme in the TAG assay kit (10010303; Cayman) and incubated at 37°C for 30 min. The absorbance at 540 nm was monitored.

#### Live cell imaging

Cells were plated on glass-bottomed 35 mm Mattek dish and transfected with desired plasmids the next day. After 1 d, cells with or without 50 μM etomoxir pretreatment for 2 h were treated with 100 μM OA (29557; CAYMAN) or same amount of BSA control (29556; CAYMAN) for 18–24 h if imaging LDs. 48 h after transfection, Cells were incubated with BODIPY 493/503 (#D3922; Thermo Fisher Scientific) at 1 μg/ml or MitoTracker Green (M7514; Thermo Fisher Scientific) at 75–100 nM for 30 min, washed with PBS, and subjected to further live cell imaging in 1× live cell imaging solution (A14291DJ; Thermo Fisher Scientific). For the 30-min OA treatment, the cells were treated with 100 μM OA for 30 min before staining and imaging.

At the Center for Cellular and Molecular Imaging Facility at Yale, imaging was performed with a 63× oil-immersion objective on an inverted Zeiss LSM 880 laser scanning confocal microscope with AiryScan, using Zen Black acquisition software (RRID: SCR_018163; https://research.unityhealth.to/wp-content/uploads/2015/09/ZEN-Black-Quick-Guide.pdf). It has a 63× oil-immersion objective lens with NA = 1.4. Z-stack images were taken. Images obtained with AiryScan were first processed using Airyscan Processing in Zen Black, and all images were analyzed in Fiji (ImageJ).

For high throughput imaging of LDs, confocal microscopy was performed on Nikon Ti2-E inverted microscope with a 63× oil-immersion objective, CSUX1 camera Photometrics Prime 95B, Agilent laser 488 and 561 nm, and a temperature setting at 37 C by the Oko Lab control system. Z-stack images were taken. Images were acquired using Nikon Elements and analyzed in Fiji (ImageJ).

Different samples were imaged in one session with the same settings. All data categorizing mitochondrial morphology were scored blindly. For LDs analysis, The Z-stack was maximum projected and the images were set at the same threshold. LDs were analyzed after converting to binary.

#### Statistical analysis

Statistical analysis between groups was performed using Prism (GraphPad Software, RRID: SCR_002798; http://www.graphpad.com) with Welch’s two-tailed unpaired *t* test. Results were indicated in the following manner: ∗ for P < 0.05, ∗∗ for P < 0.01, *** for P < 0.001, ∗∗∗∗ for P < 0.0001, where P < 0.05 is considered as significantly different.

Detailed protocols are available at  https://dx.doi.org/10.17504/protocols.io.yxmvm27kbg3p/v1. Tabular data for Fig. 2, A and B (10.5281/zenodo.7149420, 10.5281/zenodo.7149052), Fig. 3 G (10.5281/zenodo.7149142), and Fig. 4 C (10.5281/zenodo.7149282) are available in ASAP.Zenodo.

Plasmids generated for this study have been deposited in Addgene and will be available under the IDs 192832, 192833, 192834, 192835, 192837, 192866, and 192867.

### Online supplemental material

Fig. S1 shows a characterization of MIGA2 mutant constructs. Fig. S2 shows lipid binding and transfer data for MIGA2. Fig. S3 shows an analysis of cellular LD and TAG contents in WT and MIGA2 mutant cells. Table S1 lists primers used in this study.

## Supplementary Material

Table S1 lists primers.

SourceData F3 contains original blots for Fig. 3.

SourceData FS2 contains original blots for Fig. S2.

SourceData FS3 contains original blots for Fig. S3.

## References

[bib1] Ashkenazy, H., S. Abadi, E. Martz, O. Chay, I. Mayrose, T. Pupko, and N. Ben-Tal. 2016. ConSurf 2016: An improved methodology to estimate and visualize evolutionary conservation in macromolecules. Nucleic Acids Res. 44:W344–W350. 10.1093/nar/gkw408 27166375 PMC4987940

[bib2] Benador, I.Y., M. Veliova, K. Mahdaviani, A. Petcherski, J.D. Wikstrom, E.A. Assali, R. Acin-Perez, M. Shum, M.F. Oliveira, S. Cinti, . 2018. Mitochondria bound to lipid droplets have unique bioenergetics, composition, and dynamics that support lipid droplet expansion. Cell Metab. 27:869–885 e866. 10.1016/j.cmet.2018.03.003 29617645 PMC5969538

[bib3] Bian, X., Y. Saheki, and P. De Camilli. 2018. Ca(2+) releases E-Syt1 autoinhibition to couple ER-plasma membrane tethering with lipid transport. EMBO J. 37:219–234. 10.15252/embj.201797359 29222176 PMC5770786

[bib4] Choudhary, V., and R. Schneiter. 2021. A unique junctional interface at contact sites between the endoplasmic reticulum and lipid droplets. Front. Cell Dev. Biol. 9:650186. 10.3389/fcell.2021.650186 33898445 PMC8060488

[bib5] Du, X., and H. Yang. 2020. Triacylglycerol measurement in HeLa cells. Bio Protoc. 10:e3852. 10.21769/BioProtoc.3852 PMC7842829 33659499

[bib6] Du, X., L. Zhou, Y.C. Aw, H.Y. Mak, Y. Xu, J. Rae, W. Wang, A. Zadoorian, S.E. Hancock, B. Osborne, . 2020. ORP5 localizes to ER-lipid droplet contacts and regulates the level of PI(4)P on lipid droplets. J. Cell Biol. 219:e201905162. 10.1083/jcb.201905162 31653673 PMC7039201

[bib7] Emsley, P., B. Lohkamp, W.G. Scott, and K. Cowtan. 2010. Features and development of Coot. Acta Crystallogr. D Biol. Crystallogr. 66:486–501. 10.1107/S0907444910007493 20383002 PMC2852313

[bib8] Freyre, C.A.C., P.C. Rauher, C.S. Ejsing, and R.W. Klemm. 2019. MIGA2 links mitochondria, the ER, and lipid droplets and promotes de novo lipogenesis in adipocytes. Mol. Cell. 76:811–825.e14. 10.1016/j.molcel.2019.09.011 31628041

[bib9] Henne, M., J.M. Goodman, and H. Hariri. 2020. Spatial compartmentalization of lipid droplet biogenesis. Biochim. Biophys. Acta Mol. Cell Biol. Lipids. 1865:158499. 10.1016/j.bbalip.2019.07.008 31352131 PMC7050823

[bib10] Horenkamp, F.A., D.P. Valverde, J. Nunnari, and K.M. Reinisch. 2018. Molecular basis for sterol transport by StART-like lipid transfer domains. EMBO J. 37:e98002. 10.15252/embj.201798002 29467216 PMC5852651

[bib11] Kabsch, W. 2010. Integration, scaling, space-group assignment and post-refinement. Acta Crystallogr. D Biol. Crystallogr. 66:133–144. 10.1107/S0907444909047374 20124693 PMC2815666

[bib12] Kim, H., S. Lee, Y. Jun, and C. Lee. 2022. Structural basis for mitoguardin-2 mediated lipid transport at ER-mitochondrial membrane contact sites. Nat. Commun. 13:3702. 10.1038/s41467-022-31462-6 35764626 PMC9239997

[bib13] Kumar, N., M. Leonzino, W. Hancock-Cerutti, F.A. Horenkamp, P. Li, J.A. Lees, H. Wheeler, K.M. Reinisch, and P. De Camilli. 2018. VPS13A and VPS13C are lipid transport proteins differentially localized at ER contact sites. J. Cell Biol. 217:3625–3639. 10.1083/jcb.201807019 30093493 PMC6168267

[bib14] Lees, J.A., M. Messa, E.W. Sun, H. Wheeler, F. Torta, M.R. Wenk, P. De Camilli, and K.M. Reinisch. 2017. Lipid transport by TMEM24 at ER-plasma membrane contacts regulates pulsatile insulin secretion. Science. 355:eaah6171. 10.1126/science.aah6171 28209843 PMC5414417

[bib15] Liebschner, D., P.V. Afonine, M.L. Baker, G. Bunkoczi, V.B. Chen, T.I. Croll, B. Hintze, L.W. Hung, S. Jain, A.J. McCoy, . 2019. Macromolecular structure determination using X-rays, neutrons and electrons: Recent developments in phenix. Acta Crystallogr. D Struct. Biol. 75:861–877. 10.1107/S2059798319011471 31588918 PMC6778852

[bib16] Maeda, K., K. Anand, A. Chiapparino, A. Kumar, M. Poletto, M. Kaksonen, and A.C. Gavin. 2013. Interactome map uncovers phosphatidylserine transport by oxysterol-binding proteins. Nature. 501:257–261. 10.1038/nature12430 23934110

[bib17] McCoy, A.J., R.W. Grosse-Kunstleve, P.D. Adams, M.D. Winn, L.C. Storoni, and R.J. Read. 2007. Phaser crystallographic software. J. Appl. Crystallogr. 40:658–674. 10.1107/S0021889807021206 19461840 PMC2483472

[bib18] Moser von Filseck, J., A. Copic, V. Delfosse, S. Vanni, C.L. Jackson, W. Bourguet, and G. Drin. 2015. INTRACELLULAR TRANSPORT. Phosphatidylserine transport by ORP/Osh proteins is driven by phosphatidylinositol 4-phosphate. Science. 349:432–436. 10.1126/science.aab1346 26206936

[bib19] Olzmann, J.A., and P. Carvalho. 2019. Dynamics and functions of lipid droplets. Nat. Rev. Mol. Cell Biol. 20:137–155. 10.1038/s41580-018-0085-z 30523332 PMC6746329

[bib20] Prinz, W.A., A. Toulmay, and T. Balla. 2020. The functional universe of membrane contact sites. Nat. Rev. Mol. Cell Biol. 21:7–24. 10.1038/s41580-019-0180-9 31732717 PMC10619483

[bib29] Ran, F.A., P.D. Hsu, J. Wright, V. Agarwala, D.A. Scott, and F. Zhang. 2013. Genome engineering using the CRISPR-Cas9 system. Nat. Protoc. 8:2281–2308. 10.1038/nprot.2013.143 24157548 PMC3969860

[bib21] Reinisch, K.M., and W.A. Prinz. 2021. Mechanisms of nonvesicular lipid transport. J. Cell Biol. 220:e202012058. 10.1083/jcb.202012058 33605998 PMC7901144

[bib22] Saheki, Y., X. Bian, C.M. Schauder, Y. Sawaki, M.A. Surma, C. Klose, F. Pincet, K.M. Reinisch, and P. De Camilli. 2016. Control of plasma membrane lipid homeostasis by the extended synaptotagmins. Nat. Cell Biol. 18:504–515. 10.1038/ncb3339 27065097 PMC4848133

[bib23] Tian, W., C. Chen, X. Lei, J. Zhao, and J. Liang. 2018. CASTp 3.0: Computed atlas of surface topography of proteins. Nucleic Acids Res. 46:W363–W367. 10.1093/nar/gky473 29860391 PMC6031066

[bib24] Tunyasuvunakool, K., J. Adler, Z. Wu, T. Green, M. Zielinski, A. Žídek, A. Bridgland, A. Cowie, C. Meyer, A. Laydon, . 2021. Highly accurate protein structure prediction for the human proteome. Nature. 596:590–596. 10.1038/s41586-021-03828-1 34293799 PMC8387240

[bib25] van Meer, G., and A.I.P.M. de Kroon. 2011. Lipid map of the mammalian cell. J. Cell Sci. 124:5–8. 10.1242/jcs.071233 21172818

[bib31] Valverde, D.P., S. Yu, V. Boggavarapu, N. Kumar, J.A. Lees, T. Walz, K.M. Reinisch, and T.J. Melia. 2019. ATG2 transports lipids to promote autophagosome biogenesis. J. Cell Biol. 218:1787–1798. 10.1083/jcb.201811139 30952800 PMC6548141

[bib26] Wang, Y., X.M. Zhou, X. Ma, Y. Du, L. Zheng, and P. Liu. 2016. Construction of nanodroplet/adiposome and artificial lipid droplets. ACS Nano. 10:3312–3322. 10.1021/acsnano.5b06852 26910792

[bib30] Xuan, Z., L. Manning, J. Nelson, J.E. Richmond, D.A. Colón-Ramos, K. Shen, and P.T. Kurshan. 2017. Clarinet (CLA-1), a novel active zone protein required for synaptic vesicle clustering and release. Elife. 6. 10.7554/eLife.29276 PMC5728718 29160205

[bib27] Zhang, X., H. Xie, D. Iaea, G. Khelashvili, H. Weinstein, and F.R. Maxfield. 2022. Phosphatidylinositol phosphates modulate interactions between the StarD4 sterol trafficking protein and lipid membranes. J. Biol. Chem. 298:102058. 10.1016/j.jbc.2022.102058 35605664 PMC9207681

[bib28] Zhang, Y., X. Liu, J. Bai, X. Tian, X. Zhao, W. Liu, X. Duan, W. Shang, H.Y. Fan, and C. Tong. 2016. Mitoguardin regulates mitochondrial fusion through MitoPLD and is required for neuronal homeostasis. Mol. Cell. 61:111–124. 10.1016/j.molcel.2015.11.017 26711011

