# Supplementary material for: Mitoguardin-2–mediated lipid transfer preserves mitochondrial morphology and lipid droplet formation
Source: J Cell Biol. 2022 Oct 25;221(12):e202207022. doi: 10.1083/jcb.202207022 (PMC9597353; doi:10.1083/jcb.202207022)
Supplement: Table S1 — lists primers. [file JCB_202207022_TableS1.docx]

**Table S1. Primers**

| CeMIGA_long_-6xhis | tttaagaaggagatatacatatgAGTATGAATCCAATTGAAGCAGAC |
| --- | --- |
|  | tggtggtggtggtgctcgagGGCGGGCAGTAGTTCGTTG |
| CeMIGA_C_-6xhis | tttaagaaggagatatacatatgATGGATGATTCATTCCGTTCTG |
|  | tggtggtggtggtgctcgagGGCGGGCAGTAGTTCGTTG |
| 6xhis-Sumo-CeMIGA_long_ | cagagaacagattggtggatccAGTATGAATCCAATTGAAGCAG |
|  | gtggtgctcgagtgcggccgcTTATAGTTCGTTGCTCATTCTTG |
| hMIGA2_long_-6xhis | tttaagaaggagatatacatatgGATGGCAACGCGGAAAGCC |
|  | tggtggtggtggtgctcgagTTGCAGCTCGCCCAGCGG |
| hMIGA2_C_-6xhis | tttaagaaggagatatacatatgGGCGACTATCCGATTCCGCTGAGC |
|  | tggtggtggtggtgctcgagTTGCAGCTCGCCCAGCGG |
| hMIGA2(170-575)-6xhis | CACCACCACCACCACCACTGAGATCCGGC |
|  | GCTGCTCGCCGCCGGCACA |
| 3xFLAG-hMIGA2_long_ | ccaggggccccttgcggccgccGATGGCAACGCGGAAAGC |
|  | agggatgccacccgggatccTTATTGCAGCTCGCCCAG |
| 3xFLAG-WT hMIGA2_long_-25xGS-6xhis | gggtggtggaagcggcggaggaggtagtcatcaccatcaccatcacTAAGGATCCCGGGTGGCATC |
|  | ccactaccgccacctccgctgccgccaccgccactaccaccgccaccTTGCAGCTCGCCCAGCGGA |
| 3xFLAG-M1 hMIGA2_long_-25xGS-6xhis | gaggataaaagcaaccagCTGTTCTTTGGCAAAGACG |
|  | acatgtcacgcaggtaTTGAACTTTCTGGTGCTTAAAG |
| 3xFLAG-M2 hMIGA2_long_-25xGS-6xhis | agtgggtcgtcaaatgGAAACCGGCCTGATGACC |
|  | acatgtcacgcaggtaTTGAACAATCTGGTGCTTATG |
| 3xFLAG-M3 hMIGA2_long_-25xGS-6xhis | gcgaagctgcactgcGTGAACCAAGCGTTCGAAG |
|  | aaatccagaacgatgtcAAAGAATCCCATGCAAACC |
| PGK-WT MIGA2-DsRed | ggtggcctgcaggtgaattcATGGCGTTTCGTCGTGCG |
|  | gatGACGTCCTCGGTGTTGTCCATTTGCAGCTCGCCCAG |
|  | ctgggcgagctgcaaATGGACAACACCGAGGACGTC |
|  | tatgggtactcgagttaCTGGGAGCCGGAGTGGCG |
| pLVX (CMV) -WT MIGA2-DsRed | cgagctcaagcttcgATGGCGTTTCGTCGTGCG |
|  | gttgtccatTTGCAGCTCGCCCAGCGG |
|  | ctgggcgagctgcaaATGGACAACACCGAGGACGTC |
|  | ttatctagagtcgcggTTACTGGGAGCCGGAGTG |
| pLVX (CMV) –MIGA2^EEED^-DsRed | ggtgaaagaaggccgtGTTCCGTGCGAGACCCTG |
|  | aaccatccggaaccatCAGCAGACGACGATCCGC |
