# Supplementary figures and images for: Mitoguardin-2–mediated lipid transfer preserves mitochondrial morphology and lipid droplet formation
Source: J Cell Biol. 2022 Oct 25;221(12):e202207022. doi: 10.1083/jcb.202207022 (PMC9597353; doi:10.1083/jcb.202207022)

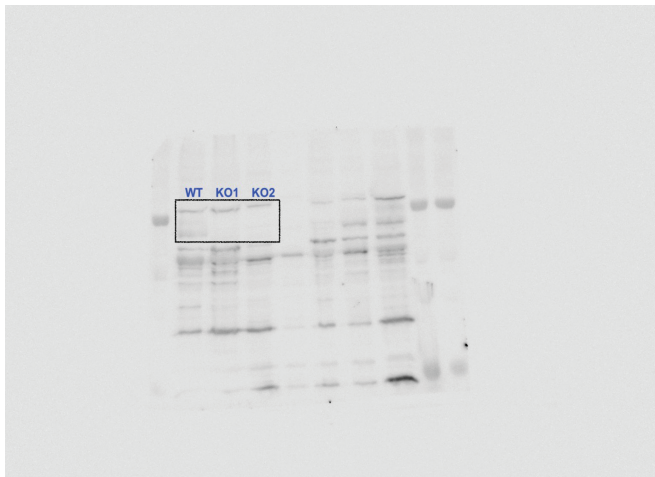

Figure 3C

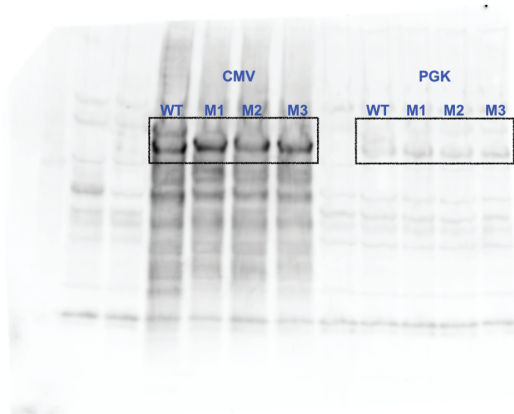

Figure 3E-upper

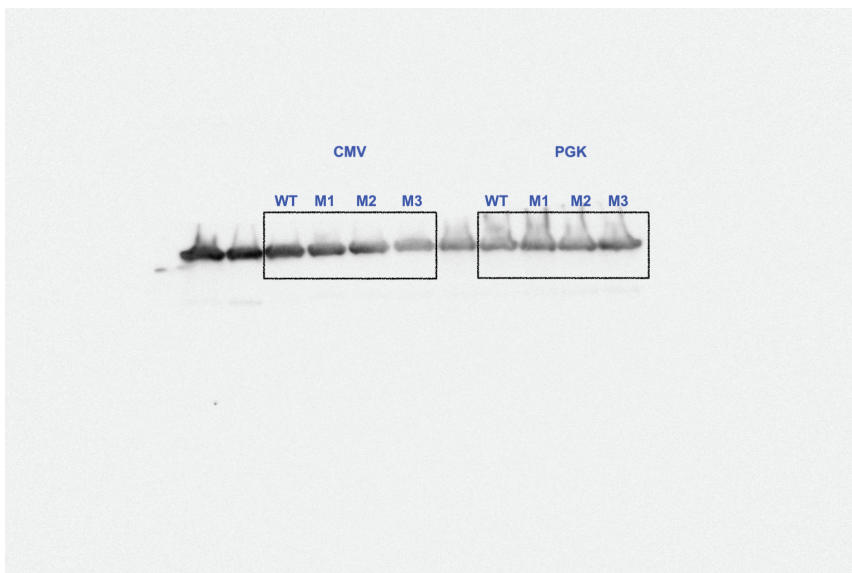

Figure 3E --bottom

Supplement: SourceData F3 — contains original blots for Fig. 3. [file JCB_202207022_SourceDataF3.pdf]

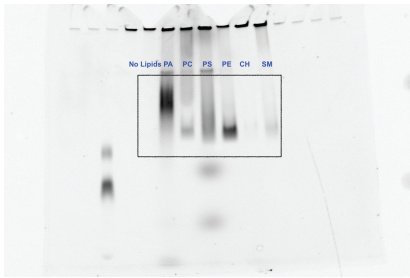

Fig S2A-upper

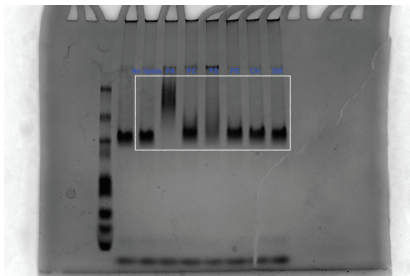

Fig S2A-bottom

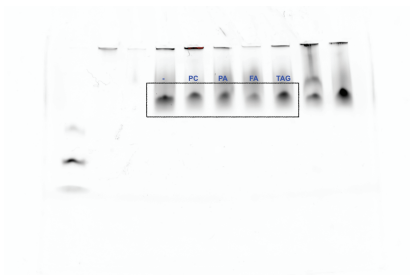

Fig S2B-upper

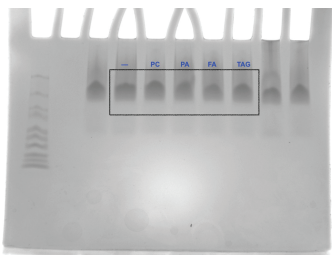

Fig S2B-bottom

Supplement: SourceData FS2 — contains original blots for Fig. S2. [file JCB_202207022_SourceDataFS2.pdf]

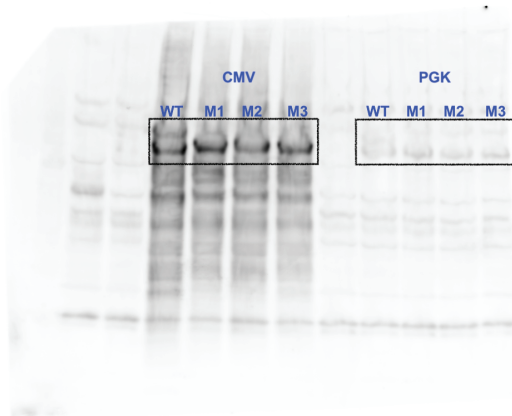

Figure S3D--upper

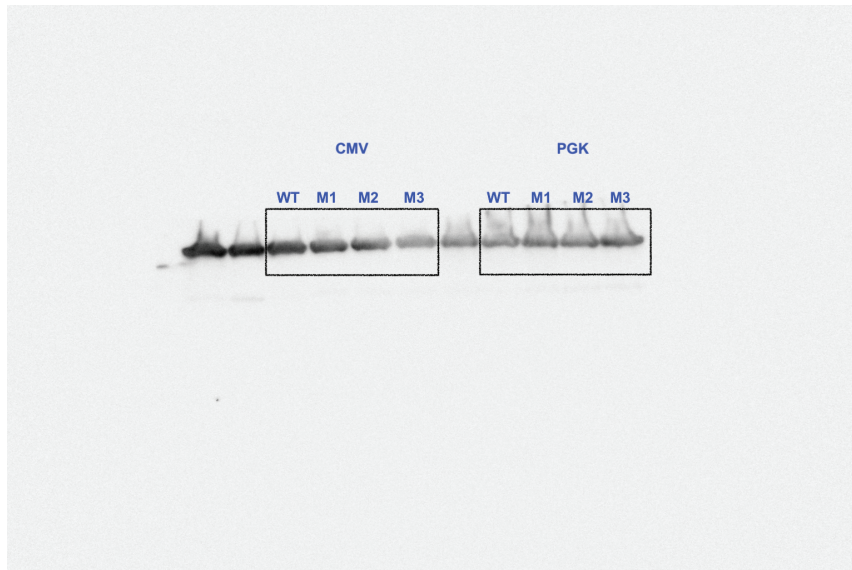

Figure S3D--bottom

Supplement: SourceData FS3 — contains original blots for Fig. S3. [file JCB_202207022_SourceDataFS3.pdf]
